# Carbon Material and Cobalt-Substitution Effects in the Electrochemical Behavior of LaMnO_3_ for ORR and OER

**DOI:** 10.3390/nano10122394

**Published:** 2020-11-30

**Authors:** Jhony X. Flores-Lasluisa, Francisco Huerta, Diego Cazorla-Amorós, Emilia Morallon

**Affiliations:** 1 Departamento Química Física e Instituto Universitario de Materiales, Universidad de Alicante, Ap. 99, E-03080 Alicante, Spain; jhony.flores@ua.es; 2 Departamento Ingenieria Textil y Papelera, Universitat Politecnica de Valencia, Plaza Ferrandiz y Carbonell, 1, E-03801 Alcoy, Spain; frahuear@txp.upv.es; 3 Departamento Química Inorgánica e Instituto Universitario de Materiales, Universidad de Alicante, Ap. 99, E-03080 Alicante, Spain

**Keywords:** cobalt-substitution, LaMnO_3_ perovskite, carbon materials, oxygen reduction reaction, oxygen evolution reaction

## Abstract

LaMn_1−x_Co_x_O_3_ perovskites were synthesized by a modified sol-gel method which incorporates EDTA. These materials’ electrochemical activity towards both oxygen reduction (ORR) and oxygen evolution reactions (OER) was studied. The cobalt substitution level determines some physicochemical properties and, particularly, the surface concentration of Co and Mn’s different oxidation states. As a result, the electroactivity of perovskite materials can be tuned using their composition. The presence of cobalt at low concentration influences the catalytic activity positively, and better bifunctionality is attained. As in other perovskites, their low electrical conductivity limits their applicability in electrochemical devices. It was found that the electrochemical performance improved significantly by physically mixing with a mortar the active materials with two different carbon black materials. The existence of a synergistic effect between the electroactive component and the carbon material was interpreted in light of the strong carbon–oxygen–metal interaction. Some mixed samples are promising electrocatalysts towards both ORR and OER.

## 1. Introduction

Research efforts on devices for the electrochemical conversion and storage of energy have multiplied in the last years due to the need for finding alternative, sustainable energy sources [1]. The two fundamental electrochemical reactions involving molecular oxygen (i.e., oxygen reduction (ORR) and oxygen evolution (OER) reactions) are of key interest for that purpose, but they both show slow reaction kinetics on conventional electrode surfaces [2,3]. At present, noble metal-based electrocatalysts remain the benchmark materials for ORR and OER, although their high cost has stimulated the search for new catalysts made of more abundant elements that would give rise to low-priced devices in the near future [4,5].

Recently, transition metal-based compounds have been considered a great alternative to developing electrocatalysts for both oxygen reactions because they are cheap and easy to synthesize [6,7]. Transition metals can adopt different oxidation states, which is crucial for electro-catalytic applications and often provide conductivity and stability [8]. Therefore, compounds such as metal oxides [9,10,11,12], metal sulfides [13], noble metal/transition metal hybrids [14,15], and bimetallic compounds [16] were reported to offer excellent performance towards ORR and OER.

In this context, perovskite-structured ABO_3_ oxides constitute an interesting option because tailoring their chemical composition results in tunable physicochemical and electrochemical properties. Unfortunately, perovskites exhibit low chemical stability in acid medium, and for that reason, alkaline solutions seem the most attractive alternative in performing both ORR and OER [17]. Alkaline media provide, on the one hand, fewer problems associated with corrosion or other forms of electrode stability, and, in addition, the oxygen-related kinetics may be more favorable in these solutions.

Lanthanum-based perovskites, LaBO_3_, are among the most studied bifunctional materials for ORR and OER [18,19,20,21]. According to literature data, the most representative B site cations are 3d transition metals and, particularly, manganese and cobalt. It is reported that partial replacement of manganese centers by cobalt in LaMnO_3_ perovskite results in a modified material, LaMn_1−x_Co_x_O_3_, with higher catalytic activity towards the target reactions [22,23,24]. The mechanism of this positive effect is, probably, twofold. First, cobalt’s addition transforms the crystal structure from either cubic or orthorhombic to rhombohedral, thereby increasing the crystallite size [25,26]. Second, an equilibrium of different oxidation states of cobalt (II and III) and manganese (III and IV) is settled [25]. As suggested [27,28], for a suitable electroactivity of perovskites, the *e_g_* orbitals of the B-site cations should be filled with one electron, an optimum value that provides a moderate interaction between surface B-site cations, molecular oxygen, and their reaction intermediates. The introduction of cobalt contributes to the filling of *e_g_* orbital and enhances electroactivity [29,30], but these materials still exhibit low electrical conductivity that affects the overall electro-catalytic performance. Such a drawback was overcome by supporting perovskites on suitable conducting substrates like carbon materials [31,32,33], which constitute excellent constituents thanks to their high-surface-area, chemical stability, and intrinsic electrical conductivity. The presence of carbon material enhances perovskites’ activity towards both ORR and OER [34,35,36]. Notably, the mechanism of ORR on carbon-modified catalysts is altered significantly. It involves reducing molecular oxygen at the carbon fraction through a two-electron transfer pathway to yield peroxide, and this latter species is then reduced to hydroxide by the perovskite component [37]. As a result, both catalytic constituents’ interaction facilitates a synergistic effect that makes these hybrids more interesting materials for ORR and OER than the separate components [34,38,39]. In addition, the overall electro-catalytic performance seems to be influenced by the physicochemical features of the carbon component, as derived from the results reported for carbon black [40], graphene [23], N-doped carbon [36], and multiwalled carbon nanotubes [41]. Apart from electrical conductivity and BET surface area, the electrochemical activity of carbon material may depend on parameters such as crystal structure or nature of catalytic sites [42,43].

In the present work, a systematic study on the electrochemical performance of LaMn_1−x_Co_x_O_3_ perovskites towards ORR and OER was carried out. A detailed study on the synthesis and characterization of these materials has been described previously [13]. More symmetrical structures can be produced if two different chelating agents are employed for the synthesis. In addition, to enhance the intrinsic electrical conductivity, perovskites are mixed, using a simple methodology, with two different carbon materials that significantly improve ORR and OER’s electroactivity. Some insights about the perovskite–carbon material interaction are proposed to explain the observed improvement.

## 2. Experimental

### 2.1. Materials and Reagents

Reagents used in this investigation were commercial Vulcan XC-72R (Vulcan) (Cabot Corporation, Billerica, MA, USA), CD-6008 carbon black (CD) (Columbian Chemicals, Brunswick, OH, USA), potassium hydroxide (KOH) (VWR Chemicals, Prague, Czech Republic), isopropanol 99.5% (Acros Organics, New Jersey, USA), Nafion^®^ 5% *w/w* water and 1-propanol (Alfa Aesar, Kandel, Germany), 20 wt % Pt/Vulcan (Sigma-Aldrich, St. Louis, MO, USA), lanthanum (III) nitrate hexahydrate (La(NO_3_)_3_·6H_2_O) (Sigma-Aldrich, St. Louis, MO, USA 99.99%), manganese (II) nitrate hydrate (Mn(NO_3_)_2_·xH_2_O) (Alfa Aesar, Kandel, Germany 99.98%), cobalt (II) nitrate hexahydrate (Co(NO_3_)_2_·6H_2_O) (Sigma-Aldrich, ACS reagent, St. Louis, MO, USA), citric acid (Sigma-Aldrich 99%, St. Louis, MO, USA), ethylenediaminetetraacetic acid (EDTA) (Sigma-Aldrich, ACS reagent, St. Louis, MO, USA), and ammonia (NH_3_) (VWR Chemicals, analytic reagent). The solutions were prepared in ultrapure water (18 MΩ/cm from an Elga Labwater Purelab system). The gases, N_2_ (99.999%), O_2_ (99.995%), H_2_ (99.999%), and synthetic air were provided by Air Liquide, and they were used without any further treatment.

### 2.2. Synthesis of Catalysts

The LaMn_1−x_Co_x_O_3_ perovskite materials were synthesized using a previously reported sol-gel method [13]. EDTA, citric acid, La(NO_3_)_3_·6H_2_O, and the sum of Mn(NO_3_)_2_·xH_2_O, and Co(NO_3_)_2_·6H_2_O were mixed using a 2:3:1:1 molar ratio, respectively. EDTA was dissolved first in a solution containing deionized water and NH_3_ at a 12.5:1 ratio. Then, citric acid and the metal precursors were added to the solution under stirring, and the pH was adjusted up to 9 with NH_3_ to form a stable sol complex. This solution was stirred at 80 °C for 6 h and then dried at 150 °C overnight on the stove. The resulting solid sample was heated at 500 °C for 30 min, and, finally, the product was ground and calcined at 700 °C for 6 h to form the perovskite materials.

LaMn_1−x_Co_x_O_3_ perovskites were physically mixed with the different carbon materials in an agate mortar for 10 min, being the simplest mechanochemistry approach [44]. A catalytic ink was then prepared by sonicating a suspension of the electrocatalyst, 1 mg mL^−1^, with 20 vol % isopropanol, 80 vol % water, and 0.02 vol % Nafion^®^ as a solvent. LaMn_1−x_Co_x_O_3_ perovskite/carbon materials were also prepared to introduce both materials in a small vial and shook by hand (this sample was named LaMn_0.7_Co_0.3_O_3_ + Vulcan) to observe the role of the agate mortar mixing in material preparation.

### 2.3. Characterization Techniques and Electrode Preparation

The surface area of LaMn_1−x_Co_x_O_3_ perovskites materials and carbon materials was obtained by physical adsorption of N_2_ (−196 °C), employing an automatic adsorption system Autosorb-6 and an Autosorb Degasser from Quantachrome Instruments (Boynton Beach, FL, USA). The samples were outgassed at 250 °C under vacuum for 8 h. The nitrogen adsorption results were used to calculate Branauer–Emmett–Teller (BET) surface values. The carbon materials’ BET surface area was 255 and 605 m^2^/g for Vulcan and CD, respectively.

Electrochemical measurements were done at 25 °C, controlled by a thermostatic bath, in a three-electrode cell in 0.1 M KOH solution using an Autolab PGSTAT302 potentiostat (Metrohm, The Netherlands). A rotating ring-disk electrode (RRDE) from Pine Research Instruments (Durham, NC, USA) equipped with a glassy carbon (GC) disk (5.61 mm diameter) and an attached Pt ring were used as working electrodes. A graphite bar was the counter electrode, and the reference electrode was a reversible hydrogen electrode (RHE) immersed in the same electrolyte.

Electroactive materials were deposited by dropping the catalytic ink on the glassy carbon disk. For perovskites, 100 µL of the dispersion was deposited, and a uniform catalyst layer of 400 µg/cm^2^ active material was obtained. For perovskites mixed with carbon materials, the study of the amount of electrocatalyst was performed, and with 120 µL (480 µg/cm^2^), the highest current was reached.

Linear sweep voltammetry (LSV) at 5 mV/s experiments were performed using different rotation rates between 400 and 2025 rpm in 0.1M KOH solution. The potential of the Pt ring was kept at 1.5 V during all the measurements. The HO_2_^−^ yield and the electron transfer number, $n_{e^{-}}$, were calculated from the hydrogen peroxide oxidation at the Pt ring electrode, according to the following equations [45]:

(1) $${{\mathrm{HO}}_{2}}^{-}\left[\%\right]\;=\;200\times \frac{I_{\mathrm{ring}}/N}{I_{\mathrm{disk}}+I_{\mathrm{ring}}/N}$$

(2) $$n_{e^{-}}\;=\;\frac{4I_{\mathrm{disk}}}{I_{\mathrm{disk}}+I_{\mathrm{ring}}/N}$$

where I_disk_ and I_ring_ are the currents measured at disk and ring electrodes, respectively, and N is the collection efficiency of the ring, which was determined experimentally as 0.37.

The OER experiments were done using RRDE, and LSV was performed from 1 to 1.8 V (vs. RHE) in the N_2_ saturated atmosphere in the rotation rate of 1600 rpm at 5 mV/s 0.1 M KOH.

The surface composition of LaMn_1−x_Co_x_O_3_ perovskites and the mixed materials was investigated by X-ray photoelectron spectroscopy (XPS, Sussex, UK) in a K-Alpha of Thermo-Scientific spectrometer, equipped with an Al anode. Deconvolution of the XPS data was done with XPSPEAK41 software (version 4.1, XPSPEAK, Hong Kong) after adjusting the experimental curves to a combination of Lorentz and Gaussian functions, and a Shirley line was used as the background.

Moreover, a selected LaMn_1−x_Co_x_O_3_ perovskite and the perovskite/carbon materials were analyzed by Temperature Programmed Reduction (TPR) (5 vol % H_2_ in Ar (35 mL/min), 10 °C/min up to 950 °C) using a Micromeritics Pulse Chemisorb 2705 with a thermal conductivity detector (TCD) (Norcross, GA, USA) to deepen the perovskite–carbon material interaction.

## 3. Results and Discussion

According to previous results [25], several physicochemical properties of LaMn_1−x_Co_x_O_3_ materials depend on the cobalt substitution level. It is then expected that altering their composition results in a modified performance towards ORR and OER. The experimental strategy was to characterize a complete set of perovskites materials by cyclic voltammetry. Later, electroactivity will be evaluated specifically towards the molecular oxygen reactions using the RRDE. Results were compared to those obtained after mixing the catalytic materials with Vulcan and other carbon materials.

### 3.1. Perovskite Materials

#### 3.1.1. Electrochemical Characterization

**Figure 1:**
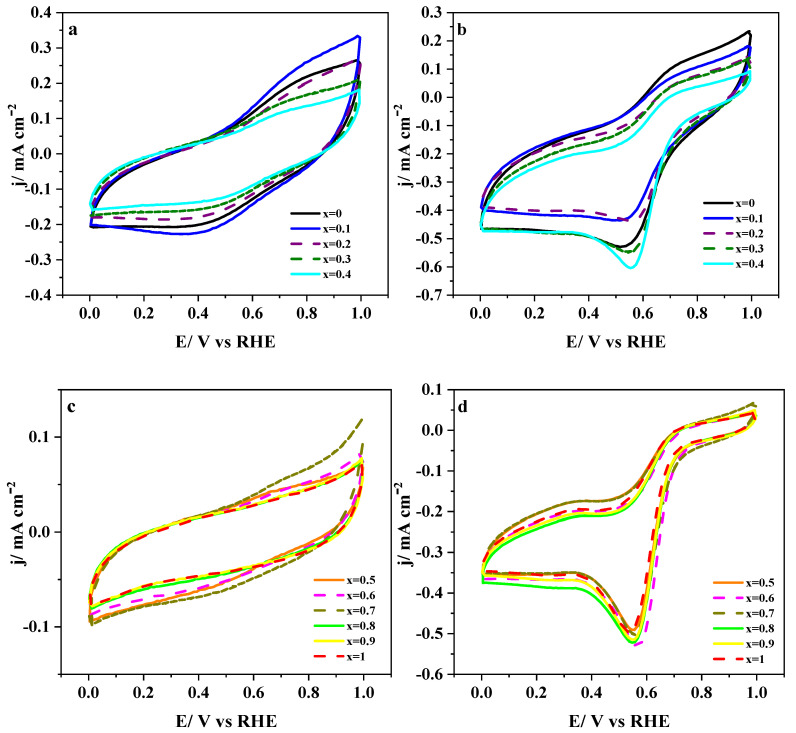
Cyclic voltammograms of LaMn_1−x_Co_x_O_3_ perovskites deposited on glassy carbon substrates in 0.1 M KOH medium saturated with either N_2_ (**a**,**c**) or O_2_ (**b**,**d**). Scan rate: 50 mV/s.

The unsubstituted LaMnO_3_ and the fully-substituted LaCoO_3_ perovskites are reported to show quite a different electrochemical behavior [18,19]. Therefore, the sequential substitution of manganese by cobalt should modify the electrochemical response of the LaMnO_3_ based materials progressively. This can be clearly observed in Figure 1a, where cyclic voltammograms for a complete set of synthesized catalysts in deoxygenated (N_2_-saturated) 0.1 M KOH medium are depicted. It can be noted that recorded current densities are overlapped with ohmic resistances caused by the metal oxides’ low electrical conductivities, and, consequently, the CVs appear slightly tilted. Most of these curves showed double-layer capacitance profiles, but faradaic contributions in the potential regions 0.6–0.9 V and 0.2–0.5 V could also be observed for materials with lower cobalt substitution (*x* < 0.5). Both charge transfer processes are related to the presence of the electrochemically active Mn^3+^/Mn^2+^ redox couple [18,46] and, accordingly, their current densities decrease as the manganese content does (Figure 1c). The cobalt redox transformation takes place at potentials beyond 1.0 V, and it cannot be observed within the potential window used in these experiments [47,48]. As reported in previous studies, both surface composition and crystal structure of LaMn_1−x_Co_x_O_3_ perovskites change sharply in the vicinity of *x* = 0.5 [25,49], which seems to be at the origin of the sudden alteration of the voltammetric profiles observed from that composition in Figure 1a.

The electrochemical behavior of perovskites in 0.1 M KOH medium saturated with O_2_ is presented in Figure 1b. The cathodic peak related to Mn^3+^ reduction cannot be clearly distinguished due to the overlapping of oxygen reduction. However, the presence of electroactive manganese was evidenced by the wide anodic peak at around 0.7 V, which, as expected, gradually disappeared by increasing the amount of cobalt. As mentioned, voltammograms are dominated by an intense oxygen reduction process that peaks at around 0.55 V and becomes more defined by increasing the cobalt substitution level (Figure 1d). In addition, the onset of O_2_ reduction shifts progressively to less positive potentials with increasing cobalt content. The shape of CVs depends noticeably on the relative cobalt content since samples with higher substitution (*x* ≥ 0.5) exhibit sharper cathodic peaks. From this result, it can be deduced that the combined physicochemical alterations [25] undergone by the LaMn_1−x_Co_x_O_3_ material in the neighborhood of *x* = 0.5 play a key role in the electroactivity towards oxygen reduction. ORR is a multistep reaction that can proceed through at least two different reaction pathways on oxygen-deficient strontium-based perovskites [50]. According to this premise, the next section will be devoted to gain more insights into the ORR mechanism at LaMn_1−x_Co_x_O_3_ materials.

#### 3.1.2. Analysis of the Electrocatalytic Activity towards ORR

**Figure 2:**
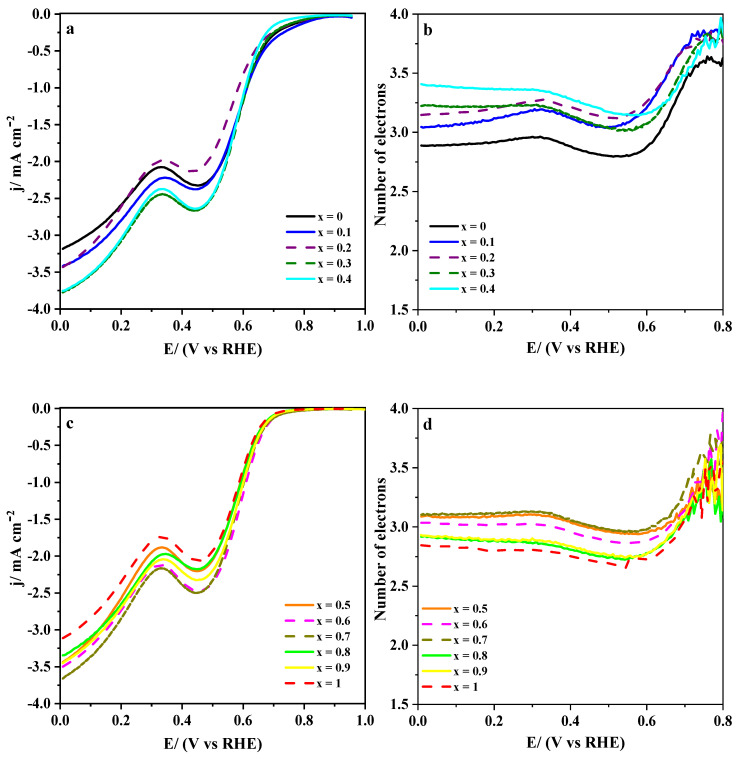
(**a**,**c**) Linear sweep voltammograms recorded at 1600 rpm for LaMn_1−x_Co_x_O_3_ perovskites in O_2_-saturated 0.1 M KOH solution. Scan rate: 5 mV/s; (**b**,**d**) Electron transfer numbers calculated from the current measured at the ring using Equation (2).

Polarization curves were recorded at a rotating ring disk electrode in 0.1 M KOH medium saturated with molecular oxygen to assess the electrochemical performance of LaMn_1−x_Co_x_O_3_ perovskites for ORR. Figure 2a,c show linear sweep voltammograms for the complete set of synthesized materials, i.e., ranging from *x* = 0 to *x* = 0.4 and from *x* = 0.5 to *x* = 1, respectively. These curves showed a first reduction process involving only oxygen that started at around 0.7 V and finished at around 0.5 V, and a second reduction process produced over oxygen and peroxide species. It extended from 0.3 V down to 0.1 V. The onset potential for oxygen reduction occurred at around 0.7 V for all samples, but it changed slightly at an increasing cobalt level. Perovskites with lower cobalt concentration (*x* < 0.5) tend to show more uniform onset potential, whereas, for the *x* ≥ 0.5 group, LaCoO_3_ is the less active material towards ORR. Figure 2b,d show the number of electrons involved in ORR. A wide range of values are recorded (2.75–3.5), but samples with cobalt content below 0.5 (Figure 2a) show slightly higher *n*_e_.

The electrochemical reactions governing the low overpotential region (roughly between 0.5–0.7 V) are the 4-electron oxygen reduction (Equation (3)) and the 2-electron oxygen reduction (Equation (4)) pathways [34,50]. Most of the peroxide generated in this latter reaction cannot undergo a further 2-electron reduction at such a moderate potential, and, consequently, it follows a chemical disproportionation reaction (Equation (5)). At a higher overpotential (~0.25 V), peroxide can be reduced to hydroxide ions (Equation (6)).

(3) $$O_{2}+2H_{2}O+\;4e^{-}⇋4{\mathrm{OH}}^{-}$$

(4) $$O_{2}+H_{2}O+\;2e^{-}⇋{\;\mathrm{HO}}_{2}^{-}+{\mathrm{OH}}^{-}$$

(5) $$2{\mathrm{HO}}_{2}^{-}⇋{\;O}_{2}+2{\mathrm{OH}}^{-}$$

(6) $${\mathrm{HO}}_{2}^{-}+H_{2}O+\;2e^{-}⇋\;3{\mathrm{OH}}^{-}$$

The 4-electron pathway is the most desirable reaction route because more power can be produced, and the generation of corrosive peroxide species is avoided [51]. When the ORR takes place on perovskites, the 4-electron pathway runs through four elementary steps, which involve the interaction of surface B-site cations and oxygen-containing species [27,39,52]: displacement of adsorbed hydroxide by oxygen molecule, surface peroxide formation, surface oxide formation, and adsorbed hydroxide regeneration. The first O_2_^2−^/OH^−^ displacement step is the rate-determining reaction and depends on the *e_g_* electron filling in B-cation. This orbital from the B-site cation interacts with the oxygen orbital and for the displacement to occur one electron has to gain sufficient energy to destabilize the B–OH^−^ bond and to form B–O_2_^2−^. However, if the *e_g_* electron filling is more than one electron, the O_2_^2−^/OH^−^ exchange does not gain sufficient energy, limiting the ORR reaction (step 1), whereas if the *e_g_* electron filling is lower than one electron, the B–O^2−^ is not sufficiently destabilized. The surface hydroxide regeneration limits the ORR kinetics (step 4) [27].

Table 1 shows a set of electrochemical parameters obtained for ORR at LaMn_1−x_Co_x_O_3_ perovskites from the LSV curves in Figure 2a. From both the onset potential and the number of electrons involved; it can be deduced that low cobalt substitution enhances these materials’ electroactivity. This finding contrasts with the fact that increasing cobalt substitution in LaMn_1−x_Co_x_O_3_ favors the presence of surface Mn^4+^ [25], a species showing higher oxidation capability than Mn^3+^. Under such conditions, peroxide chemical disproportionation (Equation (5)) should be accelerated, and, in parallel, an increase of the reaction rate of ORR through Equation (4) should be observed. However, although ORR takes advantage of the presence of Mn^4+^ species, its concentration should be low enough to facilitate the overall reaction kinetics by promoting a pathway close to 4 electrons [53]. Indeed, in Table 1, this compromise is evidenced by the fact that highly substituted samples (*x* ≥ 0.5) show a decrease in *n_e_* (while *E_onset_* stays almost constant), which is ascribed to the excess of surface Mn^4+^. Besides, the surface manganese enrichment starts to decrease for these samples, and the surface cobalt concentration is quite close to the nominal value [25]. This will result in many Co^3+^ sites, which were reported to be less active than those occupied by Mn^3+^ [18,19,27]. It was suggested [18] that the formation of the Mn^2+^/Mn^3+^ redox couple could improve the performance because the electrochemical transition is close to the ORR formal redox potential. In addition to this chemical effect, as can be concluded from the diffractogram patterns of the metal oxide materials displayed in Figure S1, the crystal structure of perovskite changes in the neighborhood of *x* = 0.5 from cubic to a less symmetric rhombohedral and, in parallel, the crystallite size enlarges. The cobalt substitution also affects the nanoparticle size of the LaMn_1−x_Co_x_O_3_ materials (Figure S2), which increases from around 25 to 60 nm with the cobalt content. As a result, the number of surface electroactive sites drops, and the current density is significantly affected.

**Figure 3:**
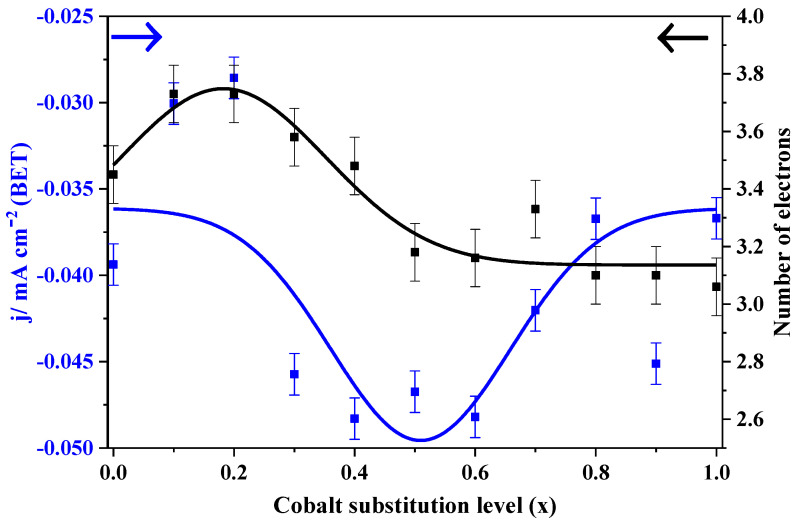
Change in BET-normalized current density and number of the electrons with increasing cobalt content in LaMn_1−x_Co_x_O_3_ perovskite materials—data obtained at 0.4 and 0.7 V, respectively.

In order to establish a relation between the cobalt substitution level in LaMn_1−x_Co_x_O_3_ perovskites and their electro-catalytic activity at 0.4 V, current densities were normalized to their respective BET surface areas, and the results are presented in Figure 3. From this plot, it can be deduced that moderate cobalt levels (mainly ranging from 0.3 to 0.6) are preferable because they provide perovskites with an improved normalized current. According to the Tafel slopes presented in Table 1, LaMn_1−x_Co_x_O_3_ materials exhibit two different ORR mechanisms. The first electron transfer seems to be the rate-determining step for samples with cobalt content below *x* = 0.5, as the Tafel slopes are higher than 110 mV decs^−1^. This figure was compatible with the formation of peroxide intermediate from adsorbed oxygen (involving two electrons), a species that could be further reduced to hydroxide.

On the contrary, for highly substituted samples (*x* ≥ 0.5), the Tafel slopes approached 90 mV dec^−1^, which represented an intermediate value suggesting a combination of the previous process and peroxide decomposition to yield HO_2_^−^ [51]. It could be then concluded that higher cobalt substitution tends to decrease Tafel slopes and enhance the electron transfer’s kinetics. However, the excess of cobalt reduces the number of catalytically active sites for dioxygen reduction through a 4-electrons pathway and shifts the onset potential to less positive values. Hence, if a compromise between all the electrochemical parameters should be reached, it seems that samples with *x* between 0.3 and 0.4 could show the best overall electro-catalytic performance.

#### 3.1.3. Analysis of the Electrocatalytic Activity towards OER

**Figure 4:**
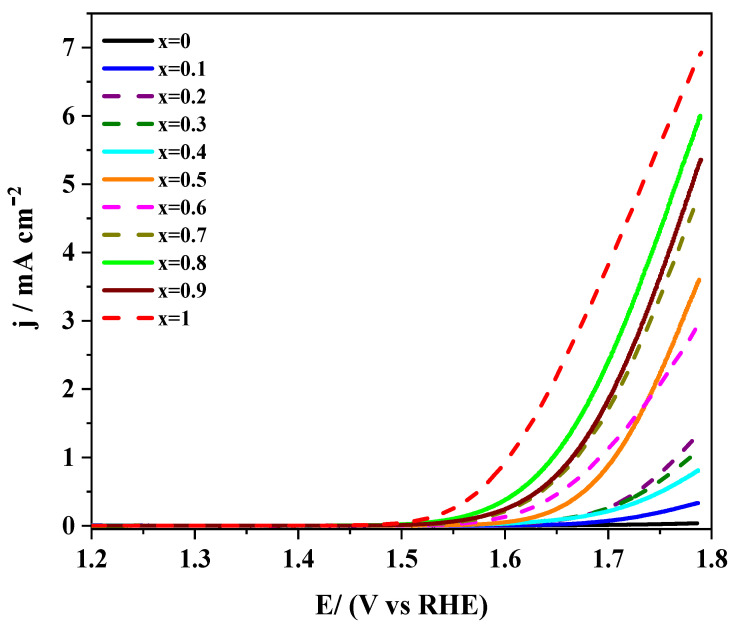
Linear sweep voltammograms recorded for LaMn_1−x_Co_x_O_3_ perovskites in RRDE in 0.1 M KOH saturated with N_2_. Scan rate 5 mV/s.

The oxygen evolution reaction can proceed through two different pathways depending on the applied potential. At low anodic overpotential, a 2-electron step transforms hydroxide into peroxide, which subsequently can disproportionate into oxygen and hydroxide. At high overpotential, the OER’s mechanism is reverted, and the formation of intermediates, such as surface peroxide (OOH^−^), are the rate-determining steps [28]. The response of LaMn_1−x_Co_x_O_3_ perovskites towards OER in 0.1 M KOH is shown in Figure 4.

It can be observed as a general tendency that catalysts’ electroactivity increases as the level of cobalt does, particularly beyond *x* = 0.5. Such a tendency is the opposite of the results presented above for ORR. This behavior can be interpreted in terms of the more appropriate *e_g_* orbital filling provided by cobalt, which increases the covalence of the B-O bond, thereby assisting the rate-determining steps [28,54]. Moreover, the surface enrichment in Mn^4+^ at higher cobalt substitution promotes the chemical disproportionation of peroxide (Equation (5)), thereby improving the catalytic activity for this reaction [55]. Some studies reported that substitution of manganese by cobalt could shorten the Mn-Mn bond distance, thereby promoting the formation of O-O bonds on the perovskite surface and avoiding peroxide formation. As a result, surface O_2_^2−^ species are directly generated, and the electroactivity of these materials improves [22,56].

Table 2 shows that the potential required to reach a given current density is less positive at increasing cobalt content. Moreover, Tafel plots reveal that samples with higher cobalt levels exhibited lower slope values, thus proving better kinetics for the electron transfer process. This effect was due to the relative strength of B–OH_ads_ bonds formed with either Mn or Co. The bond was weaker for cobalt but still strong enough to provide suitable catalytic properties [54,57]. It could be concluded that the activity towards OER increased at increasing cobalt levels.

### 3.2. Perovskite Materials Mixed with Carbon Materials

The occurrence of a synergistic effect between perovskites and carbon materials was already reported for oxygen electrocatalysis [58]. In addition to improving electrical conductivity, carbon materials can act as co-catalysts by reducing O_2_ to H_2_O_2_. This latter species can be further reduced over the adjacent perovskite sites to hydroxide either electrochemically (Equation (6)) or by disproportion (Equation (5)) [37,59,60]. However, to take advantage of this effect, the optimum mass ratio between both materials must be determined. The perovskite sample selected for optimization was LaMn_0.7_Co_0.3_O_3,_ and the carbon material used was Vulcan. The results are presented in the supplementary information for ORR. Different perovskite: carbon black relative contents were analyzed, and it was found that the 1:1 sample exhibited higher double-layer capacitance (see Figure S3). Despite that the onset potential for this sample does not differ significantly from the other mixed materials, the key difference appears in the number of electrons involved and, particularly, in the lower Tafel slope (see Figure S4 and Table S1).

#### 3.2.1. Electrochemical Characterization

**Figure 5:**
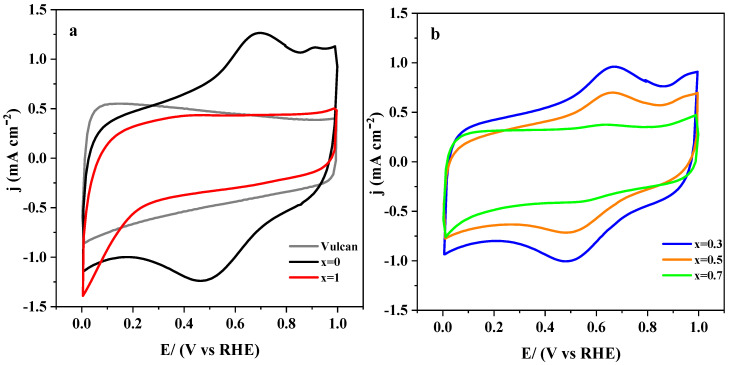
Cyclic voltammograms recorded in 0.1 M KOH medium saturated with N_2_ for the samples: (**a**) Vulcan XC-72R (light grey curve) and the LaMn_1−x_Co_x_O_3_ perovskites (x = 0 and x = 1) mixed with Vulcan (with 1:1 mass ratio) and (**b**) LaMn_1−x_Co_x_O_3_ perovskites (x = 0.3, 0.5 and 0.7) mixed with Vulcan (with 1:1 mass ratio). Scan rate 50 mV/s.

LaMnO_3_, LaCoO_3,_ and intermediate LaMn_1−x_Co_x_O_3_ perovskite materials mixed with Vulcan in the same mass ratio were characterized electrochemically and the results presented in Figure 5. The CV of the carbon material has also been included for comparison purposes. Within the potential region studied, neither the fully-substituted LaCoO_3_ nor the carbon material showed redox activity and, consequently, their respective cyclic voltammograms showed only double-layer charging processes (Figure 5a). In agreement with the results presented for the perovskites materials (see Figure 1a), manganese’s presence originates a clear reversible redox feature centered at about 0.6 V and it is ascribed to the Mn^3+^/Mn^2+^ transition. The current of this peak was significantly higher than that recorded for perovskites. As expected, the effect of increasing the cobalt substitution level (Figure 5b) is to decrease the peak intensity of the Mn^3+^/Mn^2+^ process progressively. An essential voltammetric difference of the Vulcan-mixed electrocatalysts against the self-contained metal oxides is the former’s higher electrical conductivity, which makes the voltammetric profile more symmetrical. As described above, LaMn_1−x_Co_x_O_3_ perovskites with low cobalt content tend to exhibit better performance towards ORR, whereas high cobalt levels promote OER. We checked the ability of the mixed materials to catalyze both electrochemical reactions.

#### 3.2.2. Electro-catalytic Activity towards ORR and OER

**Figure 6:**
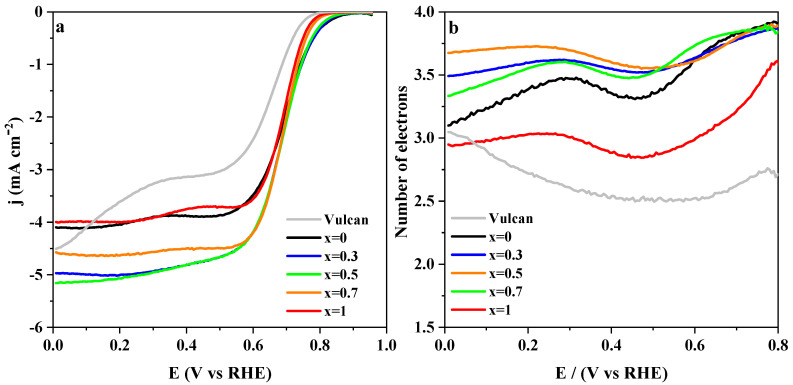
(**a**) RDE linear sweep voltammograms for LaMn_1−x_Co_x_O_3_ perovskite/Vulcan XC-72R (1:1 mass ratio) in 0.1 M KOH saturated with O_2_ at 1600 rpm; (**b**) Number of electrons involved in ORR at increasing potential as obtained from Equation (2) using the current measured at the ring.

Figure 6a shows the LSV curves recorded for LaMn_1−x_Co_x_O_3_/Vulcan materials with the same mass ratio in an oxygen-saturated test solution. Over mixed catalysts, the mechanism of O_2_ reduction was closer to 4-electrons than on perovskites (Figure 1b), as it can be observed in Figure 6b. These results suggested the existence of a certain synergistic effect between the two components of the samples. Since both LaMnO_3_ and LaCoO_3_ perovskites exhibited the lowest limiting current and the lower number of electrons, it could be deduced that partial substitution of cobalt enhanced the electroactivity of the mixed materials significantly. Such an effect was even more apparent than that observed for perovskites.

The most significant electrochemical parameters of the ORR are collected in Table 3 for samples containing Vulcan. The onset potential of this reaction was slightly more favorable at carbon-containing metal oxides than at perovskite samples. This effect was probably related to the higher electrical conductivity and the generation of H_2_O_2_ promoted by Vulcan. The carbon material’s presence clearly enhances the limiting current for each sample compared to the pristine perovskites (see Table 1). Nevertheless, the most significant difference is the number of electrons involved, which increases up to values close to 4 (compare data in Table 1 and Table 3). Regarding perovskite composition, cobalt’s presence increased the limiting current but did not significantly influence the onset potential and number of electrons. Interestingly, the Tafel slopes revealed that the ORR mechanism was influenced by changes in the cobalt concentration, as more favorable values were obtained at increasing cobalt substitution. From those data, it could be concluded that the migration of adsorbed oxygen intermediates gained relevance as the rate-determining step compared to the first electron-transfer process [61].

**Figure 7:**
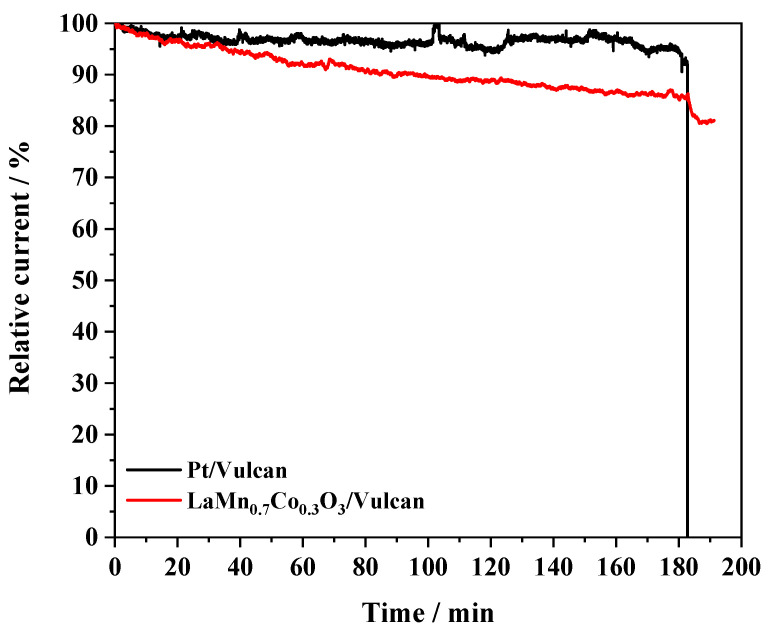
Comparative stability test for LaMn_0.7_Co_0.3_O_3_/Vulcan (1:1 mass ratio) and 20% Pt/Vulcan carried out at 0.65 V and 1600 rpm in O_2_-saturated 0.1 M KOH and 25 °C. Methanol was added 180 min after the beginning of the experiment.

A classical methanol poisoning test has been applied to study catalysts’ stability under oxygen reduction conditions [62]. Chronoamperometric experiments were carried out at 1600 rpm on an RRDE in an O_2_-saturated 0.1 M KOH medium. The performances of commercial 20% Pt/Vulcan and LaMn_0.7_Co_0.3_O_3_/Vulcan samples were compared at a constant potential of 0.65 V. After 3 h at this potential; methanol was added to the background electrolyte until 1.0 M concentration was reached. As expected, the black curve in Figure 7 reveals the platinum-based electrocatalyst’s long-term stability, for which a moderate decrease of about 5% in intensity occurred after 180 min. However, following the addition of methanol, the recorded current dropped to zero due to the severe poisoning of the active metal by the CO produced in the methanol oxidation reaction (MOR). On the contrary, the perovskite/Vulcan sample’s electroactivity test was characterized by a slow decrease of the initial activity followed by a stabilization of the current density after 160 min. The tolerance of this sample to poisoning was significant, as derived from the minor loss of activity (less than 5%) after the addition of methanol. It could be then concluded that LaMn_0.7_Co_0.3_O_3_/Vulcan showed a substantial electro-catalytic performance that makes it a promising alternative to platinum-based electrocatalysts for the ORR in alkaline solutions.

**Figure 8:**
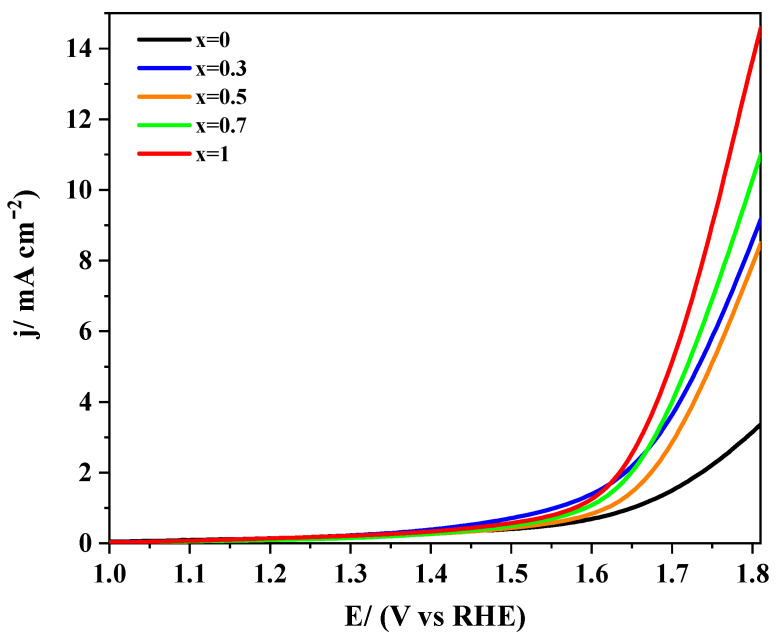
Linear sweep voltammograms recorded for LaMn_1−x_Co_x_O_3_ perovskites mixed with Vulcan in the same mass ratio in 0.1 M KOH medium saturated with N_2_. Scan rate 5 mV/s.

The response of LaMn_1−x_Co_x_O_3_/Vulcan materials with the same mass ratio towards the OER in 0.1 M KOH is presented in Figure 8. The current recorded at an anodic potential was as high as 1.8 V and it was generally higher than that recorded for the LaMn_1−x_Co_x_O_3_ perovskite materials under similar experimental conditions (Figure 4). This result suggested that the carbon material helped in releasing perovskite active sites for the reaction. The mechanism probably involved the migration of O_2_ produced at the oxide to the carbon material (spillover) [59]. On the other hand, it could be observed that cobalt-containing samples exhibited higher OER currents than the unsubstituted LaMnO_3_ perovskite. This result could be due to the lower activity of manganese itself and, besides, to the lower surface concentration of Mn (IV) species resulting from the lack of cobalt promoter [13].

The electrochemical parameters obtained for Vulcan containing samples towards the OER are collected in Table 2. It is worth noting that Tafel slopes were higher than the slopes recorded for pristine perovskites, but they followed a similar tendency. These data confirmed that cobalt improved the OER reaction kinetics. Among the samples studied, LaMn_0.7_Co_0.3_O_3_ perovskite required slightly lower potentials to yield the same amount of molecular oxygen, particularly at a high production rate. This result and the sample’s high activity towards the ORR makes the LaMn_0.7_Co_0.3_O_3_/Vulcan material a promising electrocatalyst for both reactions.

#### 3.2.3. Effect of the Carbon Materials on the ORR Electrocatalysis

**Figure 9:**
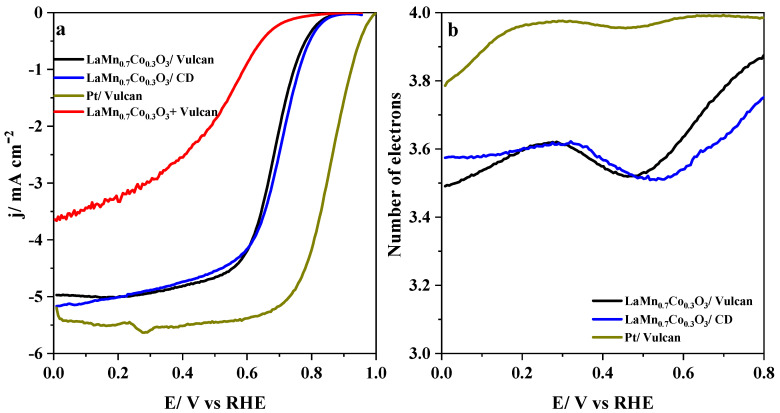
(**a**) Linear sweep voltammograms recorded at 1600 rpm on an RDE for a LaMn_0.7_Co_3_O_3_ perovskite mixed with different carbon materials (1:1 mass ratio) in an agate mortar and the sample prepared via shaking by hand in 0.1 M KOH saturated with O_2_. (**b**) Electron number calculated from the current measured at the ring.

A high surface area carbon black (CD) was also evaluated to deepen the carbon material’s influence on the electro-catalytic activity of perovskites. Since the CD material exhibited a quite similar ORR performance to Vulcan (Figure S5), the reduction should be produced by a 2 + 2 electron pathway. The two carbon black samples were mixed with LaMn_0.7_Co_0.3_O_3_ perovskite at the same mass ratio. Table 4 shows the electrochemical parameters obtained for the ORR at the pristine carbon materials and LaMn_0.7_Co_0.3_O_3_/carbon materials (the LSV curves are shown in Figure 9).

The catalysts obtained by mixing carbon materials and perovskites showed a substantial enhancement of the electro-catalytic performance compared to the pristine perovskite samples. This improvement was not observed when the materials were mixed via shaking by hand (see sample LaMn_0.7_Co_0.3_O_3_ + Vulcan in Figure 9a). This was particularly significant when key parameters such as limiting current or onset potential were considered. Despite the higher surface area of CD compared to Vulcan, the resulting electro-catalytic performance was very similar. Both carbon materials act as efficient supports, increasing both the catalyst’s electrical conductivity and the number of active sites due to the high dispersion of supported perovskites. In addition, the presence of carbon seems to trigger a synergistic effect between the two components that, together with the active role of carbon material as co-catalyst, improve the overall electro-catalytic performance in the ORR.

Moreover, to prove that the enhancement in the electro-catalytic performance is not caused by any possible change in the structure of the LaMn_0.7_Co_0.3_O_3_ perovskite using the agate mortar to prepare the LaMn_0.7_Co_0.3_O_3_/carbon materials, the mixed materials were analyzed by XRD and compared to the LaMn_0.7_Co_0.3_O_3_ perovskite. The mixed materials displayed the same XRD pattern as the pure perovskite material (Figure S6), showing that the perovskite’s crystallite size is maintained during the synthesis. The nanoparticle size of the LaMn_0.7_Co_0.3_O_3_ perovskite was not affected by mixing them with the carbon materials, as can be observed in Figure S7 with nanoparticles of around 30 nm. Thus, the ORR performance enhancement might be related to a possible interaction between both materials, which boosted the catalytic activity.

#### 3.2.4. XPS Characterization

The positive enhancement when mixing LaMn_0.7_Co_0.3_O_3_ perovskite with different carbon materials, apart from the two advantages provided by the carbon material such as the improvement of the electrical conductivity and the role of co-catalyst in ORR, might also be related to a synergistic effect between both materials facilitated by a possible interaction between them.

The electronic properties of LaMn_0.7_Co_0.3_O_3_ perovskite mixed with the same mass ratio with the different carbon materials were investigated using X-ray photoelectron spectroscopy to detect any interaction between the metal oxide and carbon materials that could provide information about the improvement of the carbon-containing perovskite materials in the ORR. According to previous studies [40,63], a C–B–O bond (B = Mn or Co) can be formed when the perovskite/carbon materials are synthesized in-situ. This process enhances the electro-catalytic response of the material towards the oxygen molecule reactions; however, a possible interaction between both materials when they are mixed physically with an agate mortar cannot be discarded.

**Figure 10:**
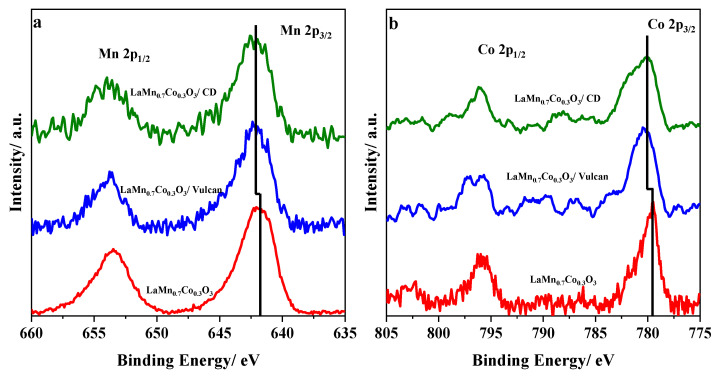
X-ray photoelectron signals obtained from (**a**) Mn 2p and (**b**) Co 2p spectral regions for LaMn_0.7_Co_0.3_O_3_ and LaMn_0.7_Co_0.3_O_3_/carbon materials with the same mass ratio.

Figure 10 displays the Mn 2p and Co 2p spectra of the LaMn_0.7_Co_0.3_O_3_/carbon materials compared to the LaMn_0.7_Co_0.3_O_3_ perovskite material, and we can observe that in all cases, a positive shift of around 0.4 eV in the Mn 2p_3/2_ and Co 2p_3/2_ peaks compared to the LaMn_0.7_Co_0.3_O_3_ perovskite is observed. This could be a consequence of a strong interaction created between the carbon material and the perovskite, which can displace the electron cloud from the metallic cations to the lighter elements, thereby increasing the binding energy of the Mn 2p and Co 2p [64]. This fact might be related to the formation of the C–O–B interaction between both materials, which facilitates the electron transfer and increases the mixed materials’ electroactivity. This shift is observed in the two carbon materials studied, demonstrating that this strong interaction occurs due to the physical mixing with the agate mortar.

To further analyze the perovskite/carbon material interaction, the O1s XPS spectra for the perovskites and the perovskites mixed with Vulcan are presented. Figure S8 shows the O 1s core-level spectra collected for a set of LaMn_0.7_Co_0.3_O_3_ perovskites (Figure S8a) and the LaMn_0.7_Co_0.3_O_3_/Vulcan plus the Vulcan support (Figure S8b). The deconvolution of the O 1s spectra of the perovskites materials results in three peaks at 529, 531, and 533 eV associated with lattice oxide species, O^2−^, surface adsorbed oxygen (O^−^, O_2_^−^ or O_2_^2−^), and oxygen-containing groups such as OH^−^, H_2_O, and chemisorbed oxygen, respectively [33,65,66].

Figure S5b shows that Vulcan carbon black exhibits two peaks at around 532.2 eV and 533.6 eV related to C=O and C–O and O–C=O bonds, respectively [67], although some contribution from adsorbed water cannot be discarded. Regarding the Vulcan containing samples, in addition to the lattice oxygen peak, a clear signal located at around 532.3 eV is observed, which can be deconvoluted into four different contributions related to the perovskite component, the carbon material, and the interaction between both materials. First, the main peak at around 532.0 eV can be associated mostly with C=O bonds of the carbon material, although oxygen-containing groups coming from perovskite at 531 eV (colored in green) are overlapping. Finally, the peak at around 533.2 eV (colored in light blue) is associated with the C–O–B interaction [40,63] produced between the Vulcan and perovskite components. In addition, the C=O signal at 532.2 eV coming from the carbon material shifts towards lower binding energy concerning the pristine material as a result of the interaction.

#### 3.2.5. TPR Characterization

The samples were characterized by the TPR technique to deepen the interaction between LaMn_0.7_Co_0.3_O_3_ perovskite and carbon materials caused by the mechanical force produced by mixing them with the agate mortar (one of the simplest mechanochemical methods). Moreover, to observe the mixing method’s effect, the same samples were prepared via shaking by hand in a small vial (these samples were named with the letters NM at the end of the nomenclature).

**Figure 11:**
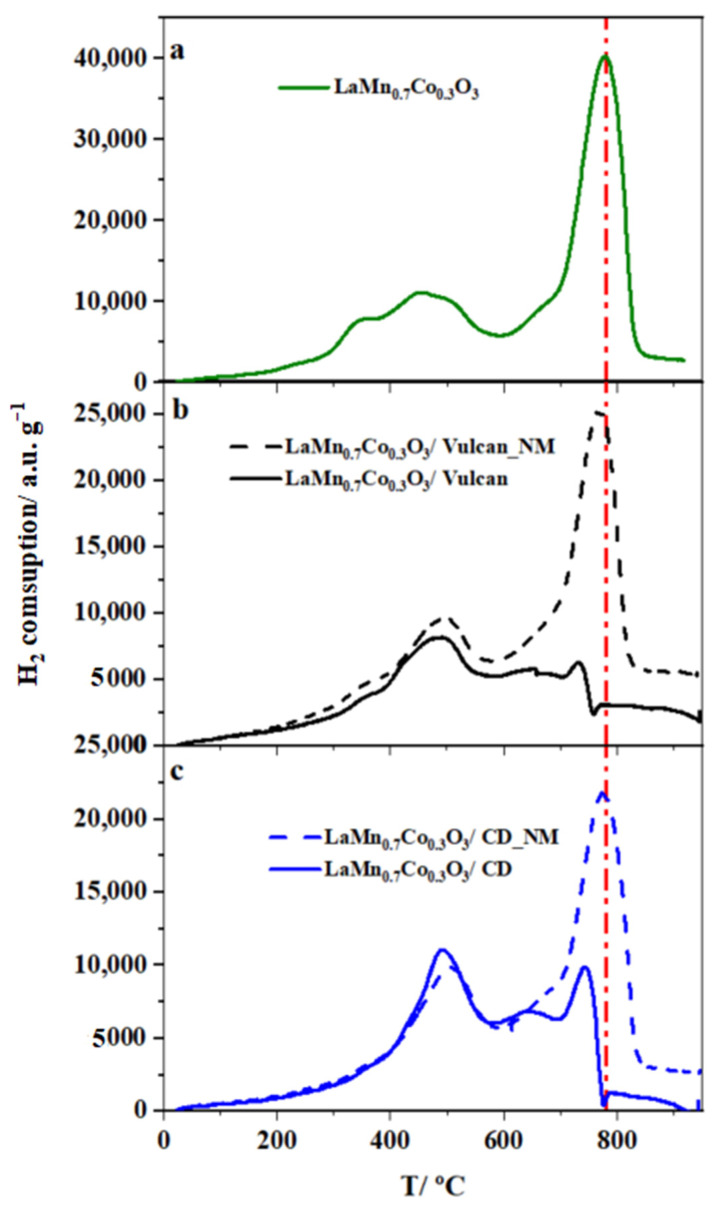
Temperature Programmed Reduction (TPR) profiles for the different mixtures prepared with a 1:1 mass ratio with (solid line) or without (dashed line) the agate mortar. (**a**) LaMn_0.7_Co_0.3_O_3_ perovskite, (**b**) LaMn_0.7_Co_0.3_O_3_/Vulcan mixtures, and (**c**) LaMn_0.7_Co_0.3_O_3_/CD mixtures.

The TPR profiles of LaMn_0.7_Co_0.3_O_3_/carbon material samples (1:1 mass ratio) together with the pure perovskite are displayed in Figure 11. Important differences can be observed depending on the mixing procedure employed. In the bulk LaMn_0.7_Co_0.3_O_3_ perovskite profile (Figure 11a), we can observe two different regions. The first region between 150–600 °C can be due to different processes such as the removal of adsorbed oxygen over the surface (150–250 °C) and the reductions of Mn^4+^ to Mn^3+^ and Co^3+^ to Co^2+^, which are overlapped in the temperature range of 250–550 °C [65]. The second region between 600–850 °C consists of a small shoulder at 650 °C related to the reduction of Co^2+^ to Co^0^ and a large reduction peak at 780 °C, corresponding to Mn^3+^ to Mn^2+^ reduction [68].

Comparing the bulk perovskite profile to the carbon-containing materials, we observed that the profiles are very similar to those for the materials mixed by shaking by hand. It revealed that the perovskite–carbon material interaction was negligible. As previously reported, some differences were observed in the low-temperature region due to some hydrogen consumption by the carbon material [69]. However, great differences could be observed when the mixture was prepared with the agate mortar, especially in the high-temperature region in which the hydrogen consumption associated with the Mn^3+^/Mn^2+^ reduction process drastically decreased. This indicated that the reduction of Mn^3+^ species was preferentially made by the carbon material which is a good reducing agent. This supports the existing interaction between both materials which is achieved by mechanical mixing with the agate mortar.

## 4. Conclusions

Bifunctional catalysts based on the LaMnO_3_ perovskite were applied to the ORR and OER, the most significant electrochemical reactions involving molecular oxygen. The sequential substitution of manganese by cobalt gives rise to LaMn_1−x_Co_x_O_3_ structures that can be used either as prepared or mixed with carbon materials to enhance the electrical conductivity. In our previous publication, surface and bulk alterations were reported to occur for LaMn_1−x_Co_x_O_3_ in the neighborhood of *x* = 0.5. It was observed that cobalt-doping increases the crystal size, and two different crystal structures could be distinguished that change from cubic to rhombohedral when the cobalt content is *x* ≥ 0.6. In addition, the presence of cobalt on the surface is more notorious when the cobalt content is *x* ≥ 0.5. This effect can also be observed in the cyclic voltammograms, where the shape and double-layer capacitance become like a pure LaCoO_3_ perovskite on increasing the cobalt content. These alterations also play a key role in their electroactivity towards the electrochemical reactions studied in this work.

Perovskites with low cobalt content tend to exhibit better ORR performance, whereas those with higher cobalt content show better electroactivity towards the OER. This effect is due to the major presence of active species of the B-site cations, but the overall reaction mechanism involves a multi-stage pathway with different rate-determining steps.

Perovskite materials mixed with carbon materials by a simple mechanochemical method show enhanced electro-catalytic properties for the two electrochemical reactions, in part due to the improvement of electrical conductivity and the role of the carbon material as co-catalyst. The key factor determining this improvement is the synergy between carbon materials (Vulcan and higher surface area carbon black (CD)) and perovskite through a strong C–O–B interaction, which facilitates the electron transfer makes more reducible the oxide materials. The existence of a synergistic effect between carbon materials and perovskite oxides for the ORR is strongly suggested. Hybrid materials, even at low cobalt content, show outstanding activity towards both the ORR and OER. In particular, the carbon-containing LaMn_0.7_Co_0.3_O_3_ samples combine high catalytic activity, good stability, and resistance to chemical poisoning, which makes it an interesting alternative to Pt-based catalysts. The results show that a simple and straightforward method based on physical mixing in an agate mortar can generate strong interactions with a high impact on perovskites’ electro-catalytic performance.

## Figures and Tables

**Table 1:** Onset potential, number of electrons, limiting current density at 0.4 V, Branauer–Emmett–Teller (BET) surface area, and Tafel slope obtained for the oxygen reduction reaction (ORR) at different perovskite materials.

Sample	$E_{onset}/V\;(\mathrm{at}-0.10\;\mathrm{mA}\;{\mathrm{cm}}^{-2})$	$n_{e^{-}}$ (at 0.7 V vs. RHE)	*j*/mA cm^−2^ (at 0.4 V)	*j*/A g^−1^ (at 0.4 V)	BET/m^2^ g^−1^	Tafel Slope/mV dec^−1^
LaMnO_3_	0.79	3.45	−2.2	−5.44	14	178
LaMn_0.9_Co_0.1_O_3_	0.81	3.73	−2.3	−5.69	19	214
LaMn_0.8_Co_0.2_O_3_	0.78	3.73	−2.1	−5.19	18	213
LaMn_0.7_Co_0.3_O_3_	0.78	3.58	−2.6	−6.43	14	151
LaMn_0.6_Co_0.4_O_3_	0.73	3.48	−2.5	−6.18	13	114
LaMn_0.5_Co_0.5_O_3_	0.70	3.18	−2.1	−5.19	11	90
LaMn_0.4_Co_0.6_O_3_	0.71	3.16	−2.3	−5.69	12	87
LaMn_0.3_Co_0.7_O_3_	0.71	3.33	−2.4	−5.93	14	103
LaMn_0.2_Co_0.8_O_3_	0.70	3.10	−2.0	−4.94	14	87
LaMn_0.1_Co_0.9_O_3_	0.70	3.10	−2.2	−5.44	12	88
LaCoO_3_	0.69	3.06	−1.9	−4.70	13	86

**Table 2:** Electrochemical parameters obtained for perovskite materials and mixed materials (perovskite:Vulcan with 1:1 mass ratio) tested in the oxygen evolution reaction (OER).

Sample	Potential/V (at 1 mA cm^−2^)	Potential/V (at 5 mA cm^−2^)	Tafel Slope/mV dec^−1^
LaMnO_3_	-	-	486
LaMn_0.9_Co_0.1_O_3_	-	-	200
LaMn_0.8_Co_0.2_O_3_	1.77	-	111
LaMn_0.7_Co_0.3_O_3_	1.78	-	139
LaMn_0.6_Co_0.4_O_3_	-	-	156
LaMn_0.5_Co_0.5_O_3_	1.71	-	87
LaMn_0.4_Co_0.6_O_3_	1.69	-	93
LaMn_0.3_Co_0.7_O_3_	1.67	-	97
LaMn_0.2_Co_0.8_O_3_	1.65	1.77	100
LaMn_0.1_Co_0.9_O_3_	1.66	1.78	100
LaCoO_3_	1.60	1.73	94
LaMnO_3_/Vulcan	1.65	-	365
LaMn_0.7_Co_0.3_O_3_/Vulcan	1.56	1.73	332
LaMn_0.5_Co_0.5_O_3_/Vulcan	1.62	1.75	312
LaMn_0.3_Co_0.7_O_3_/Vulcan	1.59	1.72	266
LaCoO_3_/Vulcan	1.58	1.70	240

**Table 3:** Onset potential, number of electrons, limiting current density, and Tafel slope obtained for the ORR reaction at perovskite materials mixed with Vulcan with a 1:1 mass ratio.

Sample	$E_{onset}/V\;(\mathrm{at}-0.10\;\mathrm{mA}\;{\mathrm{cm}}^{-2})$	$n_{e^{-}}\;(\mathrm{at}-0.7\;V\;\mathrm{vs}.\;\mathrm{RHE})$	$j_{lim}/\mathrm{mA}\;{\mathrm{cm}}^{-2}\;(\mathrm{at}\;0.4\;V)$	$j_{lim}/A\;g^{-1}\;(\mathrm{at}\;0.4\;V)$	Tafel Slope/mV dec^−1^
LaMnO_3_/Vulcan	0.84	3.83	−3.87	−7.97	101
LaMn_0.7_Co_0.3_O_3_/Vulcan	0.84	3.78	−4.82	−9.93	81
LaMn_0.5_Co_0.5_O_3_/Vulcan	0.83	3.80	−4.82	−9.93	80
LaMn_0.3_Co_0.7_O_3_/Vulcan	0.80	3.84	−4.50	−9.27	60
LaCoO_3_/Vulcan	0.79	3.23	−3.76	−7.75	57
Vulcan support	0.77	2.63	−3.14	−6.47	62

**Table 4:** Onset potential, number of electrons, and limiting current obtained for the ORR reaction at different carbon materials and LaMn_0.7_Co_0.3_O_3_/carbon materials (1:1 mass ratio).

Sample	$E_{onset}/V\;(\mathrm{at}-0.10\;\mathrm{mA}\;{\mathrm{cm}}^{-2})$	$n_{e^{-}}\;(\mathrm{at}\;0.7\;V)$	$j_{lim}/\mathrm{mA}\;{\mathrm{cm}}^{-2}\;(\mathrm{at}\;0.4\;V)$	$j_{lim}/A\;g^{-1}\;(\mathrm{at}\;0.4\;V)$	Tafel Slope/mV dec^−1^
Vulcan	0.77	2.63	−3.14	−6.47	62
CD	0.79	2.70	−2.87	−5.91	63
LaMn_0.7_Co_0.3_O_3_/Vulcan	0.84	3.78	−4.82	−9.93	81
LaMn_0.7_Co_0.3_O_3_/CD	0.85	3.63	−4.74	−9.76	79
Pt/Vulcan	0.98	3.99	−5.51	−11.35	60

## Supplementary Materials

The following are available online at https://www.mdpi.com/2079-4991/10/12/2394/s1, Figure S1: X-ray diffraction patterns for a set of LaMn_1−x_Co_x_O_3_ perovskites at increasing level of Co-substitution, Figure S2: TEM images for perovskite materials: (a) LaMnO_3_, (b) LaMn_0.7_Co_0.3_O_3_ and (c) LaCoO_3_. Figure S3: Cyclic voltammograms recorded for a LaMn_0.7_Co_0.3_O_3_ perovskite supported on increasing amounts of Vulcan 72X-R in 0.1 M KOH saturated with either N_2_ (a) or O_2_ (b). Scan rate 50 mV s^−1^. Figure S4: (a) Linear sweep voltammograms recorded at 1600 rpm on an RDE for a LaMn_0.7_Co_0.3_O_3_ perovskite supported on increasing amounts of Vulcan 72X-R in 0.1 M KOH saturated with O_2_. (b) Electron number calculated from the current measured at the ring. Figure S5: (a) Linear sweep voltammograms for carbon materials in 0.1 M KOH saturated with O2 at 1600 rpm; (b) Number of electrons involved in ORR. Figure S6: X-ray diffraction patterns for LaMn_0.7_Co_0.3_O_3_ and LaMn_0.7_Co_0.3_O_3_ mixed with carbon materials with the same mass ratio. Figure S7: TEM images for (a) LaMn_0.7_Co_0.3_O_3_/ Vulcan and (b) LaMn_0.7_Co_0.3_O_3_/ CD with the same mass ratio. Figure S8: High-resolution X-ray photoelectron spectra obtained from O 1s signal for: (a) unsupported LaMn_1−x_Co_x_O_3_ perovskites; (b) the same materials supported on Vulcan and the Vulcan support alone. Table S1: Onset potential, number of electrons, and Tafel slopes obtained for different relative amounts of LaMn_0.7_Co_0.3_O_3_ perovskite supported on Vulcan.

## References

[1] Xu X., Wang W., Zhou W., Shao Z. (2018). Recent Advances in Novel Nanostructuring Methods of Perovskite Electrocatalysts for Energy-Related Applications. Small Methods.

[2] Dekel D.R. (2018). Review of cell performance in anion exchange membrane fuel cells. J. Power Sources.

[3] Banham D., Ye S. (2017). Current Status and Future Development of Catalyst Materials and Catalyst Layers for Proton Exchange Membrane Fuel Cells: An Industrial Perspective. ACS Energy Lett..

[4] McCrory C.C.L., Jung S., Peters J.C., Jaramillo T.F. (2013). Benchmarking Heterogeneous Electrocatalysts for the Oxygen Evolution Reaction. J. Am. Chem. Soc..

[5] Marković N.M., Schmidt T.J., Stamenković V., Ross P.N. (2001). Oxygen Reduction Reaction on Pt and Pt Bimetallic Surfaces: A Selective Review. Fuel Cells.

[6] Chen D., Chen C., Baiyee Z.M., Shao Z., Ciucci F. (2015). Nonstoichiometric Oxides as Low-Cost and Highly-Efficient Oxygen Reduction/Evolution Catalysts for Low-Temperature Electrochemical Devices. Chem. Rev..

[7] Goswami C., Hazarika K.K., Bharali P. (2018). Transition metal oxide nanocatalysts for oxygen reduction reaction. Mater. Sci. Energy Technol..

[8] Osgood H., Devaguptapu S.V., Xu H., Cho J., Wu G. (2016). Transition metal (Fe, Co, Ni, and Mn) oxides for oxygen reduction and evolution bifunctional catalysts in alkaline media. Nano Today.

[9] Longhi M., Cova C., Pargoletti E., Coduri M., Santangelo S., Patanè S., Ditaranto N., Cioffi N., Facibeni A., Scavini M. (2018). Synergistic effects of active sites’ nature and hydrophilicity on the oxygen reduction reaction activity of Pt-free catalysts. Nanomaterials.

[10] Minguzzi A., Longoni G., Cappelletti G., Pargoletti E., Di Bari C., Locatelli C., Marelli M., Rondinini S., Vertova A. (2016). The influence of carbonaceous matrices and electro-catalytic MnO_2_ nanopowders on lithium-air battery performances. Nanomaterials.

[11] Han X., He G., He Y., Zhang J., Zheng X., Li L., Zhong C., Hu W., Deng Y., Ma T.-Y. (2018). Engineering Catalytic Active Sites on Cobalt Oxide Surface for Enhanced Oxygen Electrocatalysis. Adv. Energy Mater..

[12] Gao S., Geng K. (2014). Facile construction of Mn_3_O_4_ nanorods coated by a layer of nitrogen-doped carbon with high activity for oxygen reduction reaction. Nano Energy.

[13] Han X., Zhang W., Ma X., Zhong C., Zhao N., Hu W., Deng Y. (2019). Identifying the Activation of Bimetallic Sites in NiCo_2_S_4_@g-C _3_N_4_-CNT Hybrid Electrocatalysts for Synergistic Oxygen Reduction and Evolution. Adv. Mater..

[14] Han X., Wu X., Deng Y., Liu J., Lu J., Zhong C., Hu W. (2018). Ultrafine Pt Nanoparticle-Decorated Pyrite-Type CoS 2 Nanosheet Arrays Coated on Carbon Cloth as a Bifunctional Electrode for Overall Water Splitting. Adv. Energy Mater..

[15] Zhang Z., Li X., Zhong C., Zhao N., Deng Y., Han X., Hu W. (2020). Spontaneous Synthesis of Silver-Nanoparticle-Decorated Transition-Metal Hydroxides for Enhanced Oxygen Evolution Reaction. Angew. Chem..

[16] Han X., Ling X., Yu D., Xie D., Li L., Peng S., Zhong C., Zhao N., Deng Y., Hu W. (2019). Atomically Dispersed Binary Co-Ni Sites in Nitrogen-Doped Hollow Carbon Nanocubes for Reversible Oxygen Reduction and Evolution. Adv. Mater..

[17] Gupta S., Kellogg W., Xu H., Liu X., Cho J., Wu G. (2016). Bifunctional Perovskite Oxide Catalysts for Oxygen Reduction and Evolution in Alkaline Media. Chem. Asian J..

[18] Celorrio V., Dann E., Calvillo L., Morgan D.J., Hall S.R., Fermin D.J. (2016). Oxygen Reduction at Carbon-Supported Lanthanides: The Role of the B-Site. ChemElectroChem.

[19] Ashok A., Kumar A., Bhosale R.R., Almomani F., Malik S.S., Suslov S., Tarlochan F. (2018). Combustion synthesis of bifunctional LaMO_3_ (M = Cr, Mn, Fe, Co, Ni) perovskites for oxygen reduction and oxygen evolution reaction in alkaline media. J. Electroanal. Chem..

[20] Sunarso J., Torriero A.A.J., Zhou W., Howlett P.C., Forsyth M. (2012). Oxygen reduction reaction activity of La-based perovskite oxides in alkaline medium: A thin-film rotating ring-disk electrode study. J. Phys. Chem. C.

[21] Sun J., Du L., Sun B., Han G., Ma Y., Wang J., Huo H., Zuo P., Du C., Yin G. (2020). A bifunctional perovskite oxide catalyst: The triggered oxygen reduction/evolution electrocatalysis by moderated Mn-Ni co-doping. J. Energy Chem..

[22] Liu X., Gong H., Wang T., Guo H., Song L., Xia W., Gao B., Jiang Z., Feng L., He J. (2018). Cobalt-Doped Perovskite-Type Oxide LaMnO_3_ as Bifunctional Oxygen Catalysts for Hybrid Lithium-Oxygen Batteries. Chem. Asian J..

[23] Hu J., Wang L., Shi L., Huang H. (2015). Oxygen reduction reaction activity of LaMn_1−x_Co_x_O_3_-graphene nanocomposite for zinc-air battery. Electrochim. Acta.

[24] Lee D.U., Park M.G., Park H.W., Seo M.H., Ismayilov V., Ahmed R., Chen Z. (2015). Highly active Co-doped LaMnO_3_ perovskite oxide and N-doped carbon nanotube hybrid bi-functional catalyst for rechargeable zinc–air batteries. Electrochem. Commun..

[25] Flores-Lasluisa J.X., Huerta F., Cazorla-Amorós D., Morallón E. (2019). Structural and morphological alterations induced by cobalt substitution in LaMnO3 perovskites. J. Colloid Interface Sci..

[26] Pecchi G., Campos C., Peña O. (2009). Thermal stability against reduction of LaMn_1−y_CoyO_3_ perovskites. Mater. Res. Bull..

[27] Suntivich J., Gasteiger H.A., Yabuuchi N., Nakanishi H., Goodenough J.B., Shao-Horn Y. (2011). Design principles for oxygen-reduction activity on perovskite oxide catalysts for fuel cells and metal–air batteries. Nat. Chem..

[28] Suntivich J., May K.J., Gasteiger H.A., Goodenough J.B., Shao-Horn Y. (2011). A Perovskite Oxide Optimized for Oxygen Evolution Catalysis from Molecular Orbital Principles. Science.

[29] Safakas A., Bampos G., Bebelis S. (2019). Oxygen reduction reaction on La_0.8_Sr_0.2_Co_x_Fe_1−x_O_3-__δ_ perovskite/carbon black electrocatalysts in alkaline medium. Appl. Catal. B Environ..

[30] Zhao Y., Liu T., Shi Q., Yang Q., Li C., Zhang D., Zhang C. (2018). Perovskite oxides La_0.4_Sr_0.6_Co_x_Mn_1−x_O_3_ (x = 0, 0.2, 0.4) as an effective electrocatalyst for lithium—Air batteries. Green Energy Environ..

[31] Xu Y., Tsou A., Fu Y., Wang J., Tian J.-H., Yang R. (2015). Carbon-Coated Perovskite BaMnO_3_ Porous Nanorods with Enhanced Electrocatalytic Perporites for Oxygen Reduction and Oxygen Evolution. Electrochim. Acta.

[32] Alegre C., Modica E., Aricò A.S., Baglio V. (2018). Bifunctional oxygen electrode based on a perovskite/carbon composite for electrochemical devices. J. Electroanal. Chem..

[33] Hu J., Liu Q., Shi Z., Zhang L., Huang H. (2016). LaNiO_3_-nanorod/graphene composite as an efficient bi-functional catalyst for zinc–air batteries. RSC Adv..

[34] Mattick V.F., Jin X., White R.E., Huang K. (2019). Understanding the role of carbon in alkaline oxygen electrocatalysis: A case study on La_0.6_Sr_0.4_CoO_3-__δ_/Vulcan carbon composite electrocatalyst. Int. J. Hydrogen Energy.

[35] Liu K., Li J., Wang Q., Wang X., Qian D., Jiang J., Li J., Chen Z. (2017). Designed synthesis of LaCoO_3_/N-doped reduced graphene oxide nanohybrid as an efficient bifunctional electrocatalyst for ORR and OER in alkaline medium. J. Alloys Compd..

[36] Park H.W., Lee D.U., Park M.G., Ahmed R., Seo M.H., Nazar L.F., Chen Z. (2015). Perovskite-Nitrogen-Doped Carbon Nanotube Composite as Bifunctional Catalysts for Rechargeable Lithium-Air Batteries. ChemSusChem.

[37] Poux T., Napolskiy F.S., Dintzer T., Kéranguéven G., Istomin S.Y., Tsirlina G.A., Antipov E.V., Savinova E.R. (2012). Dual role of carbon in the catalytic layers of perovskite/carbon composites for the electro-catalytic oxygen reduction reaction. Catal. Today.

[38] Kéranguéven G., Ulhaq-Bouillet C., Papaefthimiou V., Royer S., Savinova E. (2017). Perovskite-carbon composites synthesized through in situ autocombustion for the oxygen reduction reaction: The carbon effect. Electrochim. Acta.

[39] Li T., Liu J., Jin X., Wang F., Song Y. (2016). Composition-dependent electro-catalytic activities of covalent carbon-LaMnO_3_ hybrids as synergistic catalysts for oxygen reduction reaction. Electrochim. Acta.

[40] Liu J., Jin X., Song W., Wang F., Wang N., Song Y. (2014). Facile preparation of modified carbon black-LaMnO_3_ hybrids and the effect of covalent coupling on the catalytic activity for oxygen reduction reaction. Chin. J. Catal..

[41] Alexander C.T., Abakumov A.M., Forslund R.P., Johnston K.P., Stevenson K.J. (2018). Role of the Carbon Support on the Oxygen Reduction and Evolution Activities in LaNiO_3_ Composite Electrodes in Alkaline Solution. ACS Appl. Energy Mater..

[42] Gabe A., Ruiz-Rosas R., Morallón E., Cazorla-Amorós D. (2019). Understanding of oxygen reduction reaction by examining carbon-oxygen gasification reaction and carbon active sites on metal and heteroatoms free carbon materials of different porosities and structures. Carbon N. Y..

[43] Ryabova A.S., Bonnefont A., Simonov P.A., Dintzer T., Ulhaq-Bouillet C., Bogdanova Y.G., Tsirlina G.A., Savinova E.R. (2017). Further insights into the role of carbon in manganese oxide/carbon composites in the oxygen reduction reaction in alkaline media. Electrochim. Acta.

[44] Boldyrev V.V. (1987). Mechanochemistry of inorganic solids. Thermochim. Acta.

[45] Xue Y., Miao H., Sun S., Wang Q., Li S., Liu Z. (2017). (La_1−x_Srx)_0.98_MnO_3_ perovskite with A-site deficiencies toward oxygen reduction reaction in aluminum-air batteries. J. Power Sources.

[46] Celorrio V., Calvillo L., Granozzi G., Russell A.E., Fermin D.J. (2018). AMnO_3_ (A = Sr, La, Ca, Y) Perovskite Oxides as Oxygen Reduction Electrocatalysts. Top. Catal..

[47] La Rosa-Toro A., Berenguer R., Quijada C., Montilla F., Morallón E., Vázquez J.L. (2006). Preparation and Characterization of Copper-Doped Cobalt Oxide Electrodes. J. Phys. Chem. B.

[48] Pawar S.M., Pawar B.S., Babar P.T., Ahmed A.T.A., Chavan H.S., Jo Y., Cho S., Kim J., Hou B., Inamdar A.I. (2019). Nanoporous CuCo_2_O_4_ nanosheets as a highly efficient bifunctional electrode for supercapacitors and water oxidation catalysis. Appl. Surf. Sci..

[49] Palikundwar U.A., Sapre V.B., Moharil S.V., Priolkar K.R. (2009). Local structure around Mn and Co in LaMn_1−x_ Co_x_O_3±__δ_: An EXAFS study. J. Phys. Condens. Matter.

[50] Mattick V.F., Jin X., Yang T., White R.E., Huang K. (2018). Unraveling Oxygen Electrocatalysis Mechanisms on a Thin-Film Oxygen-Deficient Perovskite La_0.6_Sr_0.4_CoO_3−__δ_. ACS Appl. Energy Mater..

[51] Zhang T., Anderson A.B. (2007). Oxygen reduction on platinum electrodes in base: Theoretical study. Electrochim. Acta.

[52] Wang Y., Cheng H. (2013). Oxygen Reduction Activity on Perovskite Oxide Surfaces: A Comparative First-Principles Study of LaMnO_3_, LaFeO_3_, and LaCrO_3_. J. Phys. Chem. C.

[53] Stoerzinger K.A., Risch M., Han B., Shao-Horn Y. (2015). Recent Insights into Manganese Oxides in Catalyzing Oxygen Reduction Kinetics. ACS Catal..

[54] Bockris J.O., Otagawa T. (1984). The Electrocatalysis of Oxygen Evolution on Perovskites. J. Electrochem. Soc..

[55] Zhao Y., Hang Y., Zhang Y., Wang Z., Yao Y., He X., Zhang C., Zhang D. (2017). Strontium-doped perovskite oxide La_1−x_Sr_x_MnO_3_ (x = 0, 0.2, 0.6) as a highly efficient electrocatalyst for nonaqueous Li-O_2_ batteries. Electrochim. Acta.

[56] Yamada I., Fujii H., Takamatsu A., Ikeno H., Wada K., Tsukasaki H., Kawaguchi S., Mori S., Yagi S. (2017). Bifunctional Oxygen Reaction Catalysis of Quadruple Manganese Perovskites. Adv. Mater..

[57] Malkhandi S., Trinh P., Manohar A.K., Manivannan A., Balasubramanian M., Prakash G.K.S., Narayanan S.R. (2015). Design Insights for Tuning the Electrocatalytic Activity of Perovskite Oxides for the Oxygen Evolution Reaction. J. Phys. Chem. C.

[58] Zhu Y., Zhou W., Shao Z. (2017). Perovskite/Carbon Composites: Applications in Oxygen Electrocatalysis. Small.

[59] Mefford J.T., Kurilovich A.A., Saunders J., Hardin W.G., Abakumov A.M., Forslund R.P., Bonnefont A., Dai S., Johnston K.P., Stevenson K.J. (2019). Decoupling the roles of carbon and metal oxides on the electro-catalytic reduction of oxygen on La_1−x_Sr _x_ CoO_3−__δ_ perovskite composite electrodes. Phys. Chem. Chem. Phys..

[60] Falcón H., Carbonio R., Fierro J.L. (2001). Correlation of Oxidation States in LaFe_x_Ni_1−x_O_3+__δ_ Oxides with Catalytic Activity for H_2_O_2_ Decomposition. J. Catal..

[61] Shinagawa T., Garcia-Esparza A.T., Takanabe K. (2015). Insight on Tafel slopes from a microkinetic analysis of aqueous electrocatalysis for energy conversion. Sci. Rep..

[62] Li Q., He R., Jensen J.O., Bjerrum N.J. (2003). Approaches and Recent Development of Polymer Electrolyte Membranes for Fuel Cells Operating above 100 °C. Chem. Mater..

[63] Zhou J., Song H., Ma L., Chen X. (2011). Magnetite/graphene nanosheet composites: Interfacial interaction and its impact on the durable high-rate performance in lithium-ion batteries. RSC Adv..

[64] Ge X., Goh F.W.T., Li B., Hor T.S.A., Zhang J., Xiao P., Wang X., Zong Y., Liu Z. (2015). Efficient and durable oxygen reduction and evolution of a hydrothermally synthesized La(Co_0.55_Mn_0.45_)_0.99_O_3−__δ_ nanorod/graphene hybrid in alkaline media. Nanoscale.

[65] Zhang C., Wang C., Zhan W., Guo Y., Guo Y., Lu G., Baylet A., Giroir-Fendler A. (2013). Catalytic oxidation of vinyl chloride emission over LaMnO_3_ and LaB_0.2_Mn_0.8_O_3_ (B = Co, Ni, Fe) catalysts. Appl. Catal. B Environ..

[66] Pargoletti E., Salvi A., Giordana A., Cerrato G., Longhi M., Minguzzi A., Cappelletti G., Vertova A. (2020). ORR in Non-Aqueous Solvent for Li-Air Batteries: The Influence of Doped MnO2-Nanoelectrocatalyst. Nanomaterials.

[67] Assumpção M.H.M.T., De Souza R.F.B., Rascio D.C., Silva J.C.M., Calegaro M.L., Gaubeur I., Paixão T.R.L.C., Hammer P., Lanza M.R.V., Santos M.C. (2011). A comparative study of the electrogeneration of hydrogen peroxide using Vulcan and Printex carbon supports. Carbon N. Y..

[68] Hyrve S.M., Regli S.K., Zubair M., Enger B.C., Lødeng R., Waller D., Rønning M. (2019). Catalytic Oxidation of NO over LaCo_1−x_B_x_O_3_ (B = Mn, Ni) Perovskites for Nitric Acid Production. Catalysts.

[69] Román-Martínez M.C., Cazorla-Amorós D., Linares-Solano A., de Lecea C.S.M. (1993). TPD and TPR characterization of carbonaceous supports and Pt/C catalysts. Carbon.

