# Supplementary material for: Carbon Material and Cobalt-Substitution Effects in the Electrochemical Behavior of LaMnO3 for ORR and OER
Source: Nanomaterials (Basel). 2020 Nov 30;10(12):2394. doi: 10.3390/nano10122394 (PMC7759965; doi:10.3390/nano10122394)
Supplement: Supplementary file 1 [file nanomaterials-10-02394-s001.pdf]

nanomaterials

MDPI

Supplementary Information

Carbon Material and Cobalt-Substitution Effects in the Electrochemical Behavior of $LaMnO_3$ for ORR and OER

Jhony Xavier. Flores-Lasluisa [1], Francisco Huerta [2], Diego Cazorla-Amorós [3,*] and Emilia Morallón [1,*]

[1] Departamento Química Física e Instituto Universitario de Materiales, Universidad de Alicante, Ap. 99, E-03080 Alicante, Spain; jhony.flores@ua.es

[2] Departamento Ingenieria Textil y Papelera, Universitat Politecnica de Valencia, Plaza Ferrandiz y Carbonell, 1, E-03801 Alcoy, Spain; frahuear@txp.upv.es

[3] Departamento Química Inorgánica e Instituto Universitario de Materiales, Universidad de Alicante, Ap. 99, E-03080 Alicante, Spain

* Correspondence: cazorla@ua.es (D.C.-A.); morallon@ua.es (E.M.)

The presence of cobalt in the $LaMn_{1-x}Co_xO_3$ perovskites can influence in the crystallographic properties of the materials. In Figure S1, observing the X-ray diffraction patterns of the oxide materials, we can distinguish two different tendencies due to the appearance of a double peak on the region of 2θ = 32–34°, demonstrating that two different crystal structures can be formed depending on cobalt content [1]. On one hand, the samples with $0 \leq x \leq 0.5$ exhibit a cubic structure, whereas on the other hand, the samples with $0.6 \leq x \leq 1$ have a rhombohedral structure. Besides, the introduction of cobalt that has a smaller ionic radius than manganese in the $LaMn_{1-x}Co_xO_3$ perovskites reduces the lattice volume. This effect can observe better in the region 46–47° for the materials with a rhombohedral structure where a slight shift towards higher angles is produced. Moreover, the crystallite size calculated with the Scherrer equation by Flores-Lasluisa et al. demonstrates that the cobalt substitution increases the crystallite size from 21 to 54 nm [2]. Then this fact can be an important parameter that can affect the electrochemical activity of the materials towards oxygen molecule reactions.

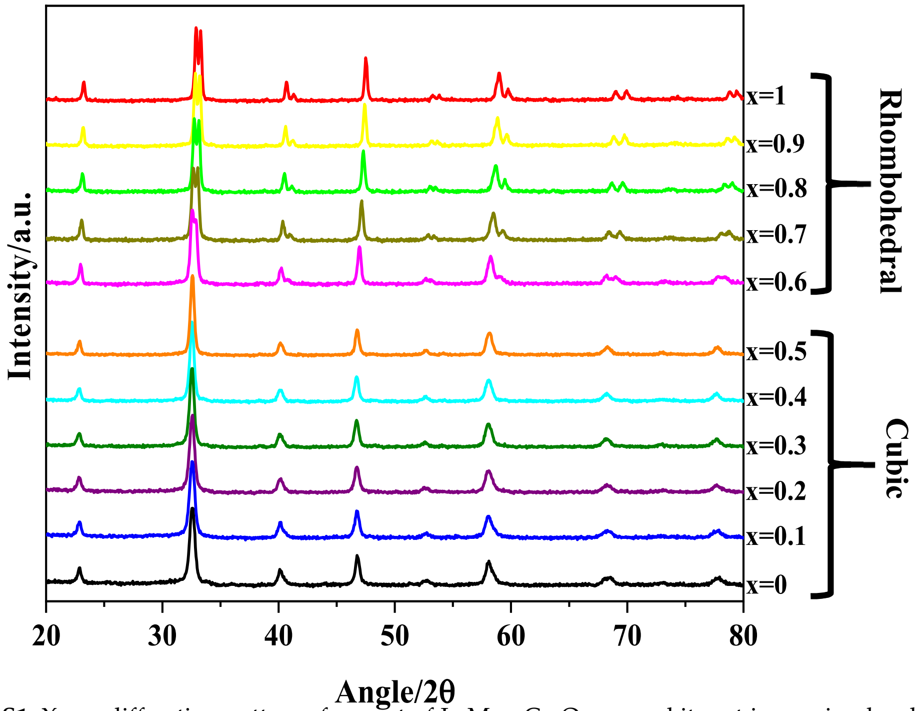

Figure S1. X-ray diffraction patterns for a set of $LaMn_{1-x}Co_xO_3$ perovskites at increasing level of Co-substitution.

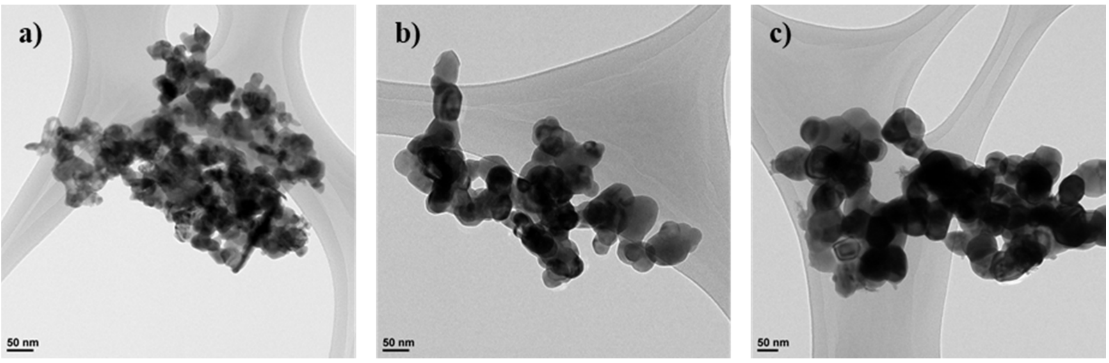

Figure S2. TEM images for perovskite materials: (**a**) $LaMnO_3$, (**b**) $LaMn_{0.7}Co_{0.3}O_3$ and (**c**) $LaCoO_3$.

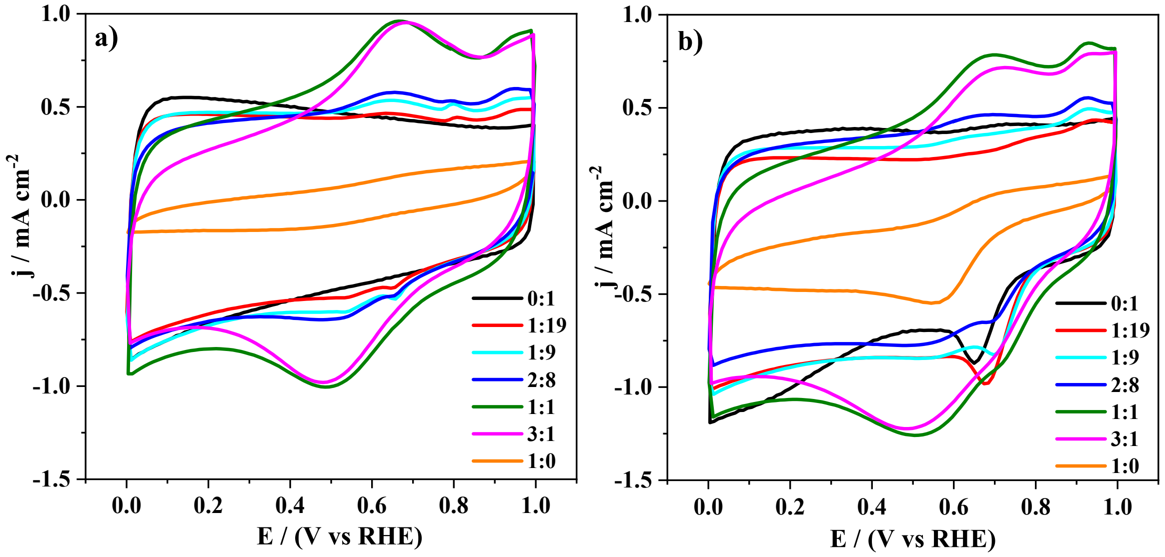

Figure S3. Cyclic voltammograms recorded for a $LaMn_{0.7}Co_{0.3}O_3$ perovskite supported on increasing amounts of Vulcan 72X-R in 0.1 M KOH saturated with either N_2 (**a**) or O_2 (**b**). Scan rate 50 mV s^{-1}.

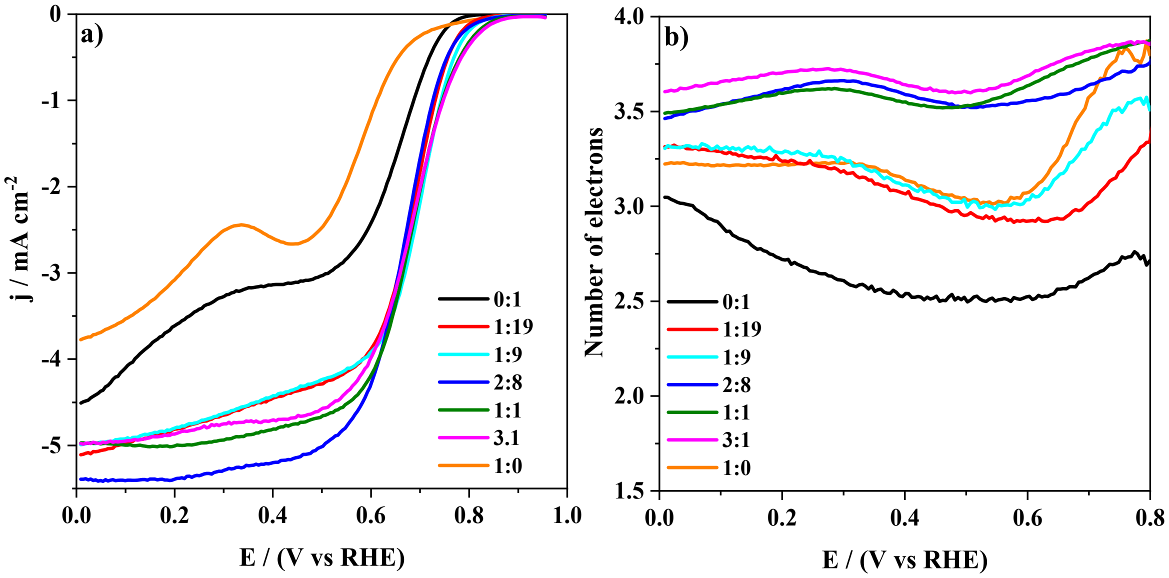

Figure S4. (**a**) Linear sweep voltammograms recorded at 1600 rpm on an RDE for a $LaMn_{0.7}Co_{0.3}O_3$ perovskite supported on increasing amounts of Vulcan 72X-R in 0.1 M KOH saturated with O_2. (**b**) Electron number calculated from the current measured at the ring.

Table S1. Onset potential, number of electrons, and Tafel slopes obtained for different relative amounts of $LaMn_{0.7}Co_{0.3}O_3$ perovskite supported on Vulcan.

Sample Perovskite:Vulcan	E_{onset} / V (at -0.10 mA cm^{-2})	n_{e^-} (at 0.7 V vs RHE)	Tafel slope / mV dec^{-1}
0:1	0.77	2.63	62
1:19	0.80	3.02	66
1:9	0.82	3.34	71
2:8	0.81	3.64	86
1:1	0.84	3.78	81
3:1	0.85	3.82	95
1:0	0.78	3.58	151

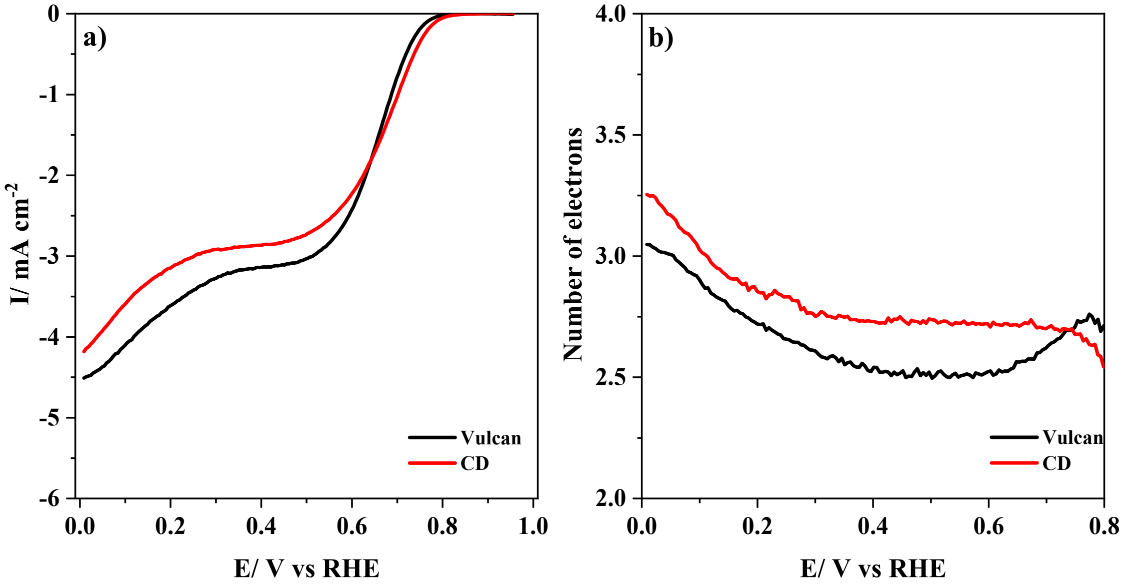

Figure S5. (**a**) Linear sweep voltammograms for carbon materials in 0.1 M KOH saturated with O_2 at 1600 rpm; (**b**) Number of electrons involved in ORR.

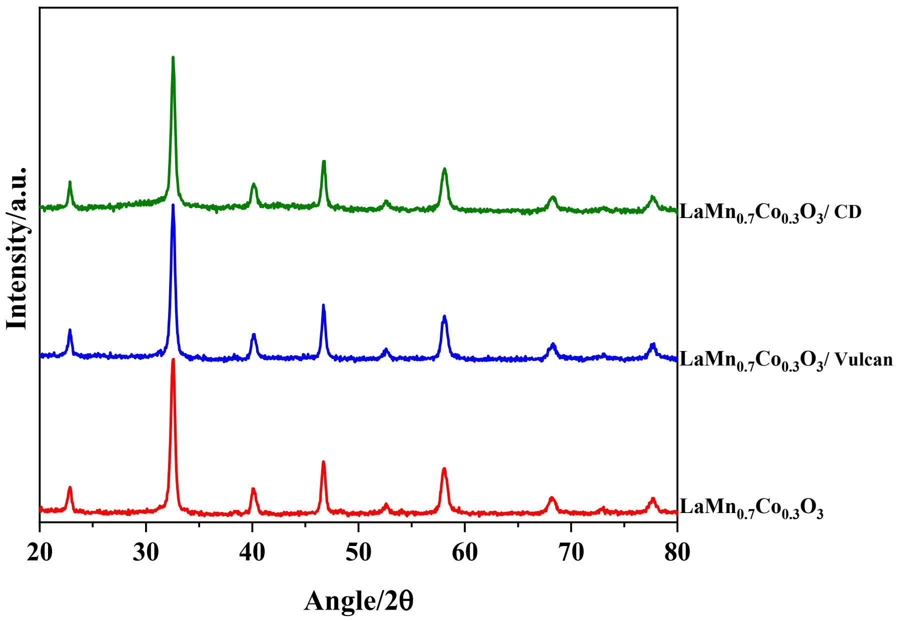

Figure S6. X-ray diffraction patterns for $LaMn_{0.7}Co_{0.3}O_3$ and $LaMn_{0.7}Co_{0.3}O_3$ mixed with carbon materials with the same mass ratio.

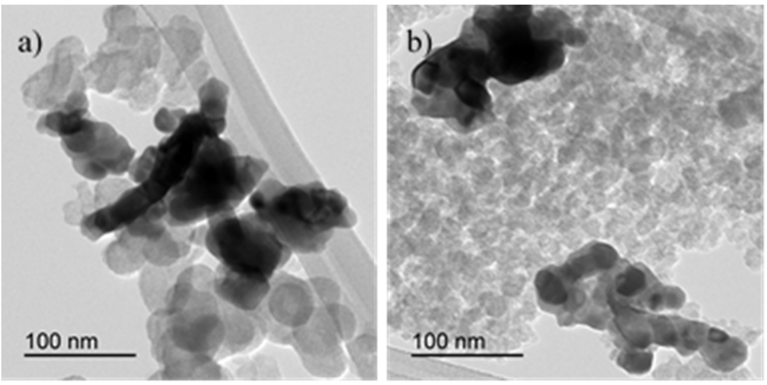

Figure S7. TEM images for (**a**) $LaMn_{0.7}Co_{0.3}O_3$/ Vulcan and (**b**) $LaMn_{0.7}Co_{0.3}O_3$/ CD with the same mass ratio.

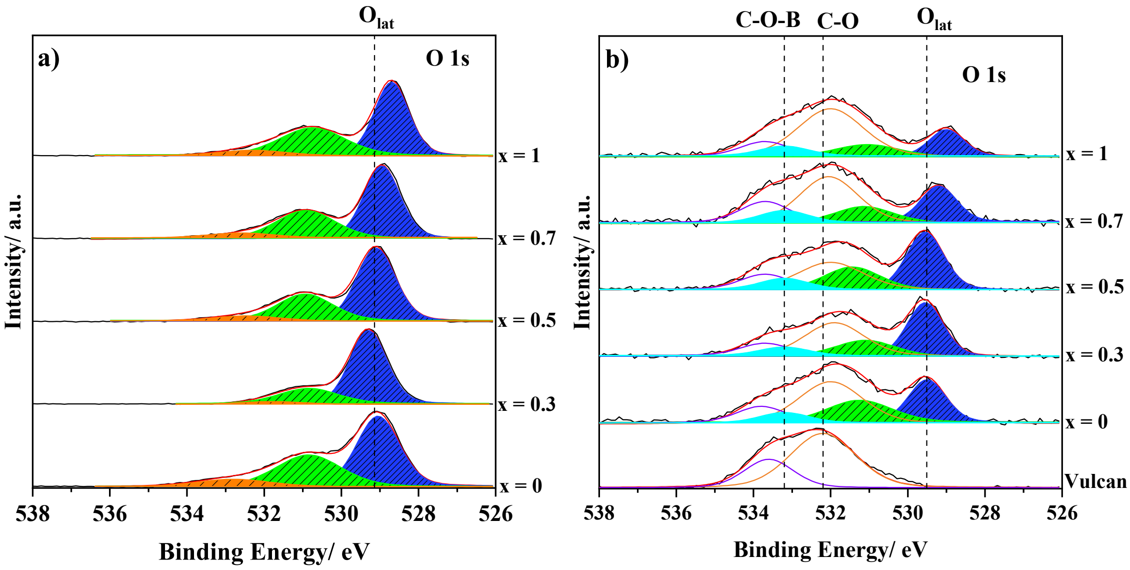

Figure S8. High-resolution X-ray photoelectron spectra obtained from O 1s signal for: **(a)** unsupported $LaMn_{1-x}Co_xO_3$ perovskites; **(b)** the same materials supported on Vulcan and the Vulcan support alone.

References Supporting

[1] M. Ao, G.H. Pham, V. Sage, V. Pareek, Structure and activity of strontium substituted $LaCoO_3$ perovskite catalysts for syngas conversion, J. Mol. Catal. A Chem. 416 (2016) 96–104. doi:10.1016/j.molcata.2016.02.020.

[2] J.X. Flores-Lasluisa, F. Huerta, D. Cazorla-Amorós, E. Morallón, Structural and morphological alterations induced by cobalt substitution in LaMnO3 perovskites, J. Colloid Interface Sci. 556 (2019) 658–666. doi:10.1016/j.jcis.2019.08.112.
